# Adaptive Time-Stepping and Machine Learning Approach for Pore-Scale Reactive Transport Simulations

**DOI:** 10.1007/s11242-026-02287-4

**Published:** 2026-02-12

**Authors:** Micha P. Baur, Sergey V. Churakov, Nikolaos I. Prasianakis

**Affiliations:** 1 Institute of Geological Sciences, University of Bern, Baltzerstrasse 1+3, Bern, 3000 Bern Switzerland; 2 PSI Center for Nuclear Engineering and Sciences, Forschungsstrasse 111, Villigen, 5232 Aargau Switzerland

**Keywords:** Claystone, Cement, Geochemical modeling, Thermodynamic modeling

## Abstract

Pore-scale reactive transport simulations are typically computationally very expensive, which limits their application to complex heterogeneous systems. To solve the computational bottleneck, an adaptive time-stepping algorithm is developed and combined with machine learning-derived surrogate models for geochemical calculations. The time-step is adapted by monitoring the evolution of the diffusion field and the precipitation reactions and by exploiting intermediate stationary states of the system. The algorithm is benchmarked on a system relevant to cement–claystone interaction with the geochemical reaction being the precipitation of Calcium-Silicate-Hydrates (C-S-H) in the pore space of a claystone. The precipitation of C-S-H is modeled as a solid solution, and it is possible to calculate and trace the local Ca/Si ratio of C-S-H, as well as the local amount of gel porosity. In the reactive transport simulations presented here, the geochemical surrogate models alone lead to acceleration factors of up to two orders of magnitude. The adaptive time-stepping algorithm leads to an additional acceleration of three to five orders of magnitude maintaining the relative deviation below 2%. The overall combined acceleration is demonstrated to be six to seven orders of magnitude having a profound impact and opening new computational avenues.

**Supplementary Information:**

The online version contains supplementary material available at https://doi.org/10.1007/s11242-026-02287-4.

## Introduction

The mineral dissolution and precipitation in porous media play an important role in natural and industrial processes, such as weathering (Yuan et al. 2017), ore deposition (Gysi and Williams-Jones 2013), metasomatism (Putnis and Austrheim 2010; Rubatto et al. 2023), CO_2_-sequestration (Kang et al. 2010; Tian and Wang 2017; Dai et al. 2020; Xie et al. 2023), radioactive waste disposal (Prasianakis et al. 2020; Rajyaguru et al. 2021; Churakov et al. 2024), concrete degradation (Patel et al. 2014, 2018; Laloy and Jacques 2022), combustion (Xu et al. 2018) and catalysis (Khatoonabadi et al. 2024). Dissolution and precipitation reactions in these systems are caused by the chemical dis-equilibrium between mineral phases and pore water (Ovaysi and Piri 2014; Gao et al. 2017; Gray et al. 2018; Prasianakis et al. 2018). The resulting dissolution or precipitation of solid phases changes the pore size and modifies the pore connectivity. The microstructure change affects the transport properties of the porous media which in turn is back-coupled to its reactivity. The strong coupling between the reactivity and transport in porous media results in a nonlinear and complex evolution of the system at different time and length scales (Seigneur et al. 2019; Baqer and Chen 2022).

Large-scale Reactive Transport Models (RTMs) for macroscopic simulations use the continuum approach, which averages the characteristics of the pore space inside a representative volume (REV) (Steefel et al. 2005; Noiriel and Soulaine 2021). The chemical reactions impact the transport properties of the media via empirical correlations between macroscopic parameters like porosity and transport parameters such as permeability or diffusivity (Hommel et al. 2018), (Molins et al. 2021). Different mathematical models exist to support such correlations like the Kozeny–Carman equation or power and exponential model functions. The Kozeny–Carman equations require information about pore space characteristics that are challenging to obtain and they do not consider the pore topology and heterogeneity (Sabo and Beckingham 2021). Furthermore, the described complexity of the interaction between chemical reactions and the evolution of the microstructure can lead to large deviations and appears to be case sensitive (Mostaghimi et al. 2016; Baqer and Chen 2022; Jenni et al. 2024). Power or exponential functions are fitted with empirical data that are challenging to obtain through experiments because of the difficulty of performing them and conducting measurements within the pores (Sabo and Beckingham 2021; Lönartz et al. 2023). The validity of these relationships is questionable, especially for the evolution scenario resulting in reduction of the porosity due to geochemical reactions (Jenni et al. 2024).

Pore-scale RTMs explicitly consider the microstructure and its temporospatial evolution and the resulting feedback on the transport properties and reactivity of porous media. Thus, they complement macroscopic simulations (Oliveira et al. 2020); they can be used to couple different temporal and spatial scales (Churakov and Prasianakis 2018; Gimmi and Churakov 2019) and derive correlations between macroscopic parameters such as porosity and diffusivity or permeability for upscaling to macroscopic RTMs (Balhoff et al. 2007; Yousefzadeh and Battiato 2017). These correlations are valid for the specific investigated system, and since they consider the exact pore space from high-resolution imaging techniques, they can be more accurate than the Kozeny–Carman equation or power and exponential functions.

The cement–claystone interface is one of the engineered systems in which strong chemical gradients between materials lead to diffusive exchange of solutes and significant evolution of porosity and pore connectivity at the interface. The evolution of the cement–claystone interface is relevant for the performance and stability of systems such as tunnels, the integrity of boreholes for the exploration of oil/gas (Pusch et al. 2016), CO2-sequestration (Wigand et al. 2009) and in the context of radioactive waste disposal (Dauzeres et al. 2010; Kosakowski and Berner 2013; Bernard et al. 2020; Watson et al. 2013).

The cement has a complex microstructure with a large variation of pore sizes. The capillary pores are the largest (0.1 – 10 μm) and contain residual water molecules between the different cement phases (Gong et al. 2014). They are not bound to any crystalline structures; thus, all molecules are transported by diffusion and advection (if a pressure gradient exists) with a low dependency on the charge and size of the molecules. C-S-H is the major cohesion producing phase in Portland cement and contains significant amount of water molecules between the calcium silicate sheets, which is called gel pores with pore sizes between 0.5 and 10 nm (Jennings et al. 2008; Gong et al. 2014). The water molecules in the gel pores are mobile and enable the diffusive mass flux of hydrated ions through the gel pores. The pores below 0.5 nm are referred to as the interlayer porosity and are characterized by very low water and ion mobility. The interlayer pores are only accessible for diffusion of cations due to the negative charged surfaces analogous to the interlayer pores of clay minerals (Wigger et al. 2018).

Claystones mainly consist of clay minerals (around 50 of the total modal composition) like smectite, vermiculite, kaolinite or illite with parts of silica phases, carbonates and sulfates. Clay minerals are sheet silicates with a layered structure and small grain sizes (smaller than 2 μm). Claystones are formed by the compaction and diagenesis of clay-rich material. This compaction leads to a preferred orientation of the flake-shaped clay mineral particles and results in a strong reduction of the macro pore space to sizes of 10–100 nm (Song et al. 2015; Wigger et al. 2018). This relatively tight structure impacts the solute pathways and the transport properties with the hydraulic conductivity being very low such that diffusion is the dominant mass transport mechanism. The tortuosity and thus diffusivity are anisotropic with higher values along the preferential orientation of the clay minerals. Most clay minerals also inherit an interlayer porosity which can make up a large amount of the total porosity volume in claystones. They are characterized by small sizes of 0.5 to 1 nm between the negatively charged clay mineral layers. Therefore, the interlayer pores are only available to diffusion for cations (Wigger et al. 2018). The pore water of the claystone macropore space has a neutral pH, high alkali concentrations and an inventory of aqueous silica.

**Fig. 1:**
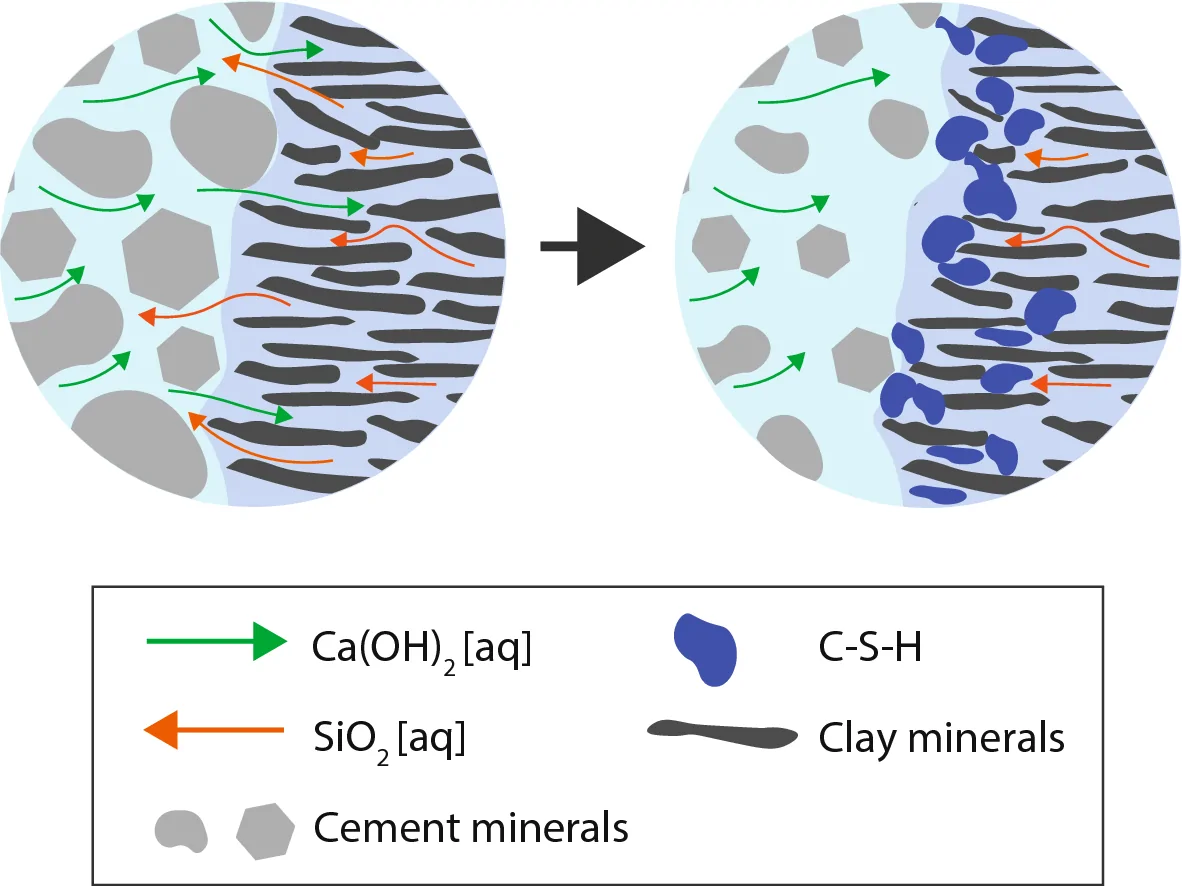
Sketch of the chemical and transport processes at the cement–claystone interface relevant for the precipitation of C-S-H. There is a diffusive flux of $\mathrm {Ca(OH)_{2\,[aq]}}$ and $\mathrm {SiO_{2\,[aq]}}$ along the interface, resulting in C-S-H precipitation at the claystone side. Adapted from (Shafizadeh 2019)

Interfaces of cement and clayrocks are common in underground engineered facilities. The corresponding materials have a very different chemistry and are prone to chemical reactivity at the interface. The pore space of the cement and claystone contains different pore water compositions at chemical equilibrium with the solid structure. This creates a geochemical gradient at the interface which is the driving force of the flux of solutes between both sides at the interface (Fig. 1, left). The mixing of pore waters results in the alteration of the local composition and as a result the pore water departs from the chemical equilibrium with the minerals of the corresponding pore. This process is responsible for the subsequent mineral precipitation and dissolution. After mixing, portlandite starts to dissolve in the cement side and the resulting $\mathrm {Ca(OH)_{2\,[aq]}}$ diffuses out into the claystone. The geochemical reactions on the claystone side are characterized by the slow dissolution of clay minerals and the precipitation of C-S-H, carbonates, zeolites and secondary clay minerals. Experiments (Shafizadeh et al. 2020), natural analogs (Savage 2011; Martin et al. 2016) and macroscopic models (Marty et al. 2014) determined Calcium-Silicate-Hydrate (C-S-H) to be one of the major precipitating solid phases on the claystone side which leads to the formation of a low porosity zone at the interface (Fig. 1, right).

Experimental observations supported the characterization of this low porosity zone which has an extent of several micro- to millimeters in the claystone side depending on the detailed mineralogy of both interface sides and the duration of the experiment (Mäder et al. 2017; Luraschi et al. 2020; Shafizadeh et al. 2020; Wilson et al. 2021). The mechanism leading to the C-S-H precipitation, the evolution of the microstructure and the effect of the gel porosity and precipitation of other mineral phases due to the solute diffusion on both sides of the interface are still under investigation. The pore space alterations at the interface affect the transport properties across this interface, and depending on the extent of the precipitation front, a significant reduction of diffusivity can be observed. For the case of nuclear waste repository, this could affect the radionuclide transport in the solute phase (leaching from the waste canister) or the gas transport originating from the corrosion of canisters or degradation of organic matter (Leupin et al. 2024). The gel porosity of C-S-H can play a key role because it is expected to remain open for the diffusion of solutes. This can be beneficial for the transport of gas molecules stemming from the corrosion of the steel containers and degradation of organic material. The possibility of gas transport is desired to prevent pressure build-up and damage to the host rock. Therefore, a better understanding of the microstructure evolution at the cement–claystone interface is important for the safety assessment of the repository.

Continuum scale simulations at the macroscopic scale of cement–clay interaction are able to provide semi-quantitative description of mineral transformation but heavily rely on empirical relationships between porosity, diffusivity and permeability. Moreover, continuum scale simulations are fundamentally unable to predict the “effective” clogging time for the interface, as it is intrinsically dependent on the discretization. Pore-scale RTM has the potential to resolve the fundamental difficulty of continuum scale modeling of such systems. However, the usage of pore-scale RTM is computationally expensive and requires realistic representation of the microstructure and the subsequent upscaling of results.

Recent advances in computational resources, numerical models and the ability to obtain realistic pore spaces by high-resolution imaging techniques (Blunt et al. 2013; Mostaghimi et al. 2016; Noiriel and Soulaine 2021) or by idealized pore structures (Mosser et al. 2017) led to an increased usage of pore-scale studies with RTMs (Molins et al. 2021). The Lattice-Boltzmann method (LBM) is a commonly used mass transport and fluid flow solver at the pore scale because it can treat complex geometries, it has simple embedding of physics and it is easy to parallelize on GPUs (Calore et al. 2016; Prasianakis et al. 2018). The coupling of LBM with geochemical solvers to obtain a reactive transport model within the pore scale is relatively new and gains more attraction (Kang et al. 2006; Zhang et al. 2022; Varzina et al. 2020; Liu et al. 2023). However, such simulations are computationally expensive which limits a wider application in realistic cases.

The calculations of chemical equilibria in RTM have to be conducted at each voxel and time-step with a geochemical solver, which are the most common computational bottleneck. Recent modeling benchmarks demonstrated that the use of surrogate models to replace geochemical solvers can speedup numerical simulations without loss in accuracy (Prasianakis et al. 2025). Such surrogate models were integrated into pore-scale models with the LBM (Prasianakis et al. 2020) or continuum models (De Lucia et al. 2017; Guérillot and Guérillot 2018; Collard et al. 2023; Demirer et al. 2023).

Surrogate models are computationally more efficient than the geochemical solvers, which leads to an overall acceleration of RTM solvers reported to be in the range of one to four orders of magnitude (Jatnieks et al. 2016; Prasianakis et al. 2020; Laloy and Jacques 2022).

Depending on the problem at hand, the mass transport solvers (e.g. LBM) require small time-steps to accurately describe the processes taking place at the micrometer scale while it is typically of interest the effect of geochemical reactions on relatively very long times. At the same time, chemical systems of interest are often characterized by slow reaction kinetics (compared to the time scale of mass transport at the micrometer scale) requiring also a fine time discretization. Under specific conditions a certain combination of slow kinetics and diffusion controlled mass transport may result in temporary quasi-steady-state transport conditions. Under such circumstances, the local diffusive flux and reaction yield reach a stationary state so that the solid alterations (e.g. precipitation or dissolution) take place at constant rates throughout the domain of interest. The change of solid volume (porosity) occurs at an even slower pace and influences the diffusive and reaction field only when significant volume changes have taken place. In other words, the system remains in that state until the dissolution/precipitation reactions significantly alter the microstructure and distort the established diffusive field.

This clear separation of time scales for mass transport and microstructure change brings the potential to apply adapted time-steps during the simulation, in order to accelerate the wall-clock time needed for the simulation. In principle, the time-step is increased when the simulated system is in a quasi-steady-state condition, where the numerical error associated with larger integration time-step remains at low levels. The concept of adaptive time-stepping has been already integrated into various numerical studies for flooding (Ruponen 2014), phase separation (Wodo and Ganapathysubramanian 2011; Zhang and Qiao 2012), reactive transport at the macroscale (Belfort et al. 2007) or blood flow (Han et al. 2021). Such methods use error estimates to calculate an optimal value for the time-step.

This paper introduces an adaptive time-stepping algorithm within the Lattice-Boltzmann framework to decrease the computational costs of RTMs at the pore scale by taking advantage of temporal quasi-steady-state conditions. The developed methodology is applied to perform 2-D pore-scale simulations of cement–claystone interaction controlled by the diffusion of $\mathrm {Ca(OH)_{2\,[aq]}}$ and $\mathrm {SiO_{2\,[aq]}}$ in the pore space of a claystone, which leads to the precipitation of Calcium-Silicate-Hydrates (C-S-H) phases. Three system parameters are introduced and tested to allow the algorithm to observe and navigate through the different system states which allow for an optimal balance between accuracy and efficiency. The results show an acceleration of the overall simulation of several orders of magnitude.

This paper is organized as follows: The geochemical system, the RTM, the machine learning-based geochemical surrogate model and the characteristic modeling parameters (effective diffusivity and Ca/Si ratio of C-S-H) are described in the chapter Methodology. The background and the algorithm of the adaptive time-stepping are elaborated in the chapter Adaptive time-stepping. It is tested and benchmarked on three systems with the results shown in chapter Results. The numerical performance of the algorithm is discussed in chapter Numerical performance with the implications and interpretation of the results on the understanding of the cement–claystone interface processes being discussed in the chapter Application of the modeling results to the cement–claystone interface reactivity.

## Methodology

### Geochemical System

The geochemical system is set up to simulate counter diffusion of $\mathrm {Ca(OH)_{2\,[aq]}}$ originating from the dissolution of portlandite on the cement side and $\mathrm {SiO_{2\,[aq]}}$ originating from the pore water of the claystone. The mixing of both solutes within the macropores results in the precipitation of C-S-H according to Eq. 1.

1 $$\begin{aligned} Ca(OH)_2 [aq] + SiO_2 [aq] \xrightarrow {/phantom{i}} (CaO)_a (SiO_2 )_b (H_2 O)_c \end{aligned}$$

The equilibrium approach is applied to calculate the volume of precipitates formed in the simulation domain at each time-step by using the geochemical solver GEMS (Kulik et al. 2013) and thermodynamic data from the PSI/Nagra 12/07 (Thoenen et al. 2014) and Cemdata18 (Lothenbach et al. 2019) database. The varying composition and structures of C-S-H are modeled by a quaternary solid solution of four endmembers (Kulik 2011) which are shown in Table 1.

**Table 1:** Information about the chemical formula composition and density of the C-S-H endmembers (Kulik 2011)

C-S-H endmember	Abbreviation	Chemical formula	Density [$g/cm^3$]
Tobermorite-D	Tob-D	$(CaO)_{5/6}(SiO_2)_{2/3}(H_2O)_{11/6}$	2.50
Tobermorite-H	Tob-H	$(CaO)_{2/3}(SiO_2)(H_2O)_{3/2}$	2.25
Jennite-D	Jen-D	$(CaO)_{3/2}(SiO_2)_{2/3}(H_2O)_{5/2}$	2.10
Jennite-H	Jen-H	$(CaO)_{4/3}(SiO_2)(H_2O)_{13/6}$	2.30

**Fig. 2:**
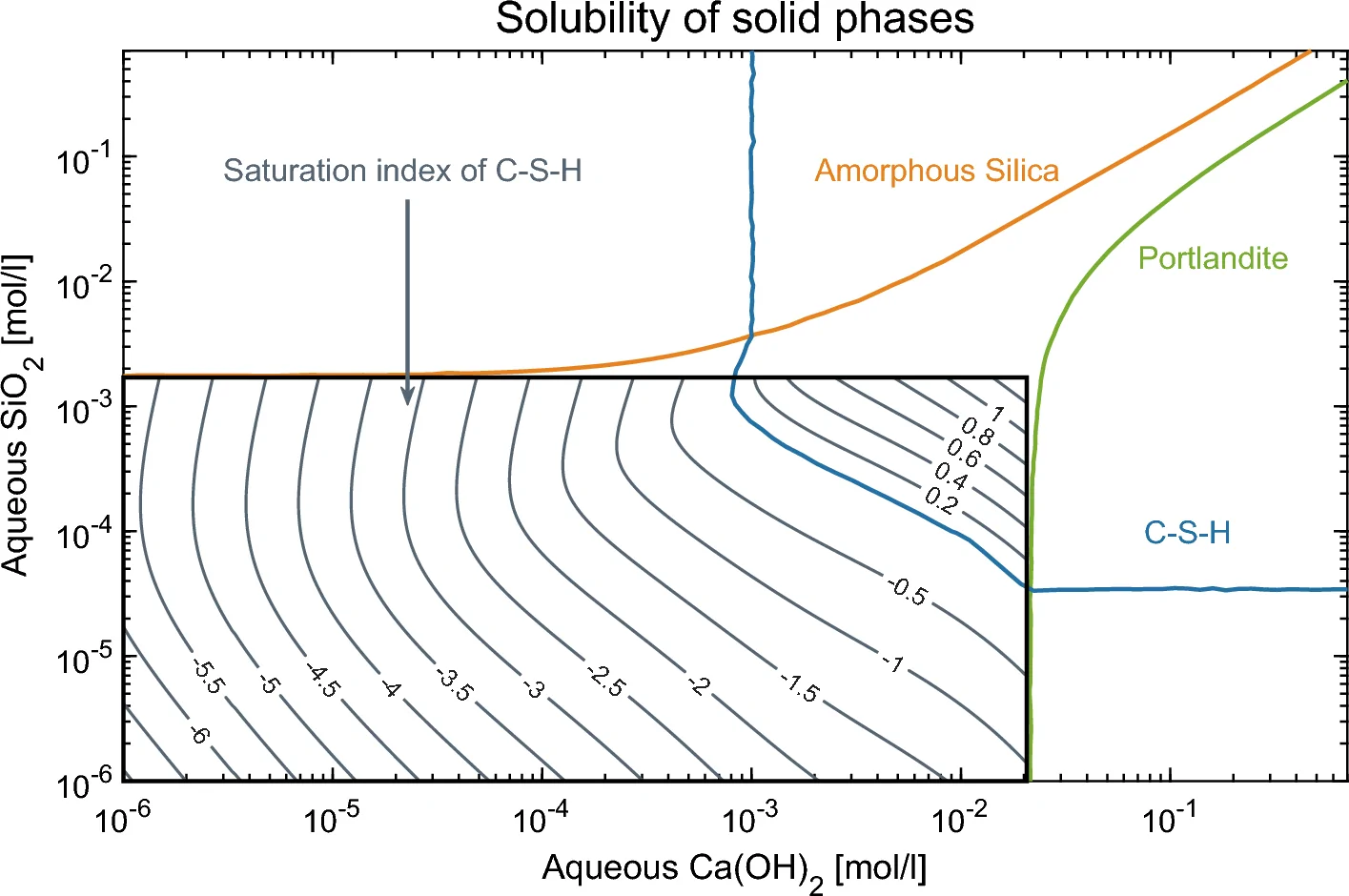
Solubility of amorphous silica (orange), portlandite (green) and C-S-H (blue). The black box displays the considered concentration range with the contour lines (gray) of C-S-H saturation indices

The relatively low supersaturation calculated from the possible solute concentrations for the cement–claystone interface favors the heterogeneous nucleation of precipitates on existing mineral surfaces, while the homogeneous nucleation mechanism can be neglected (Garrault-Gauffinet and Nonat 1999). The limiting factor of maximum concentrations of aqueous solutes is the equilibrium pore water of portlandite (0.02065 mol L^− 1^ of $\mathrm {Ca(OH)_{2\,[aq]}}$) and amorphous silica (0.0017 mol L^− 1^ of $\mathrm {SiO_{2\,[aq]}}$). C-S-H can have a maximum achievable saturation index of 1.36 below the maximum concentrations of aqueous solutes (Fig. 2). The zones in the macropores that are characterized by C-S-H precipitation will never be sufficiently undersaturated for significant dissolution of C-S-H because of the lack of other sinks for the aqueous solutes (other than precipitation of C-S-H) and the choice of the boundary conditions. Therefore, the dissolution of C-S-H is neglectable and is not considered.

### Reactive Transport Model

**Fig. 3:**
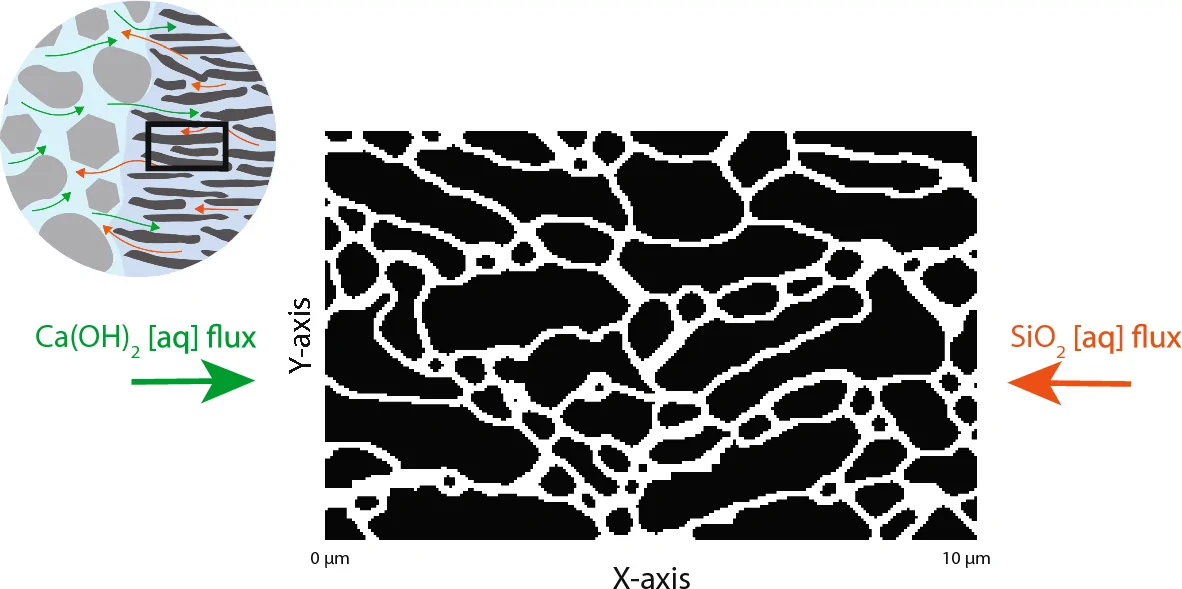
Example of a simulation domain representing the macropore space of a claystone close to the cement–claystone interface with information about the boundary conditions. The clay minerals are black, and the fully saturated macropores are white

The microstructure of the model considered for the reactive transport simulations (RT-simulations) comprises a claystone pore model consisting of irregularly shaped and chemically inert clay minerals and fully saturated macropores (Fig. 3). The underlying simulation domain is a modified section of a claystone domain constructed and presented in Gimmi and Churakov (2019). The left and right boundaries are set to simulate geochemical gradients with a diffusive flux of $\mathrm {Ca(OH)_{2\,[aq]}}$ to the right and $\mathrm {SiO_{2\,[aq]}}$ to the left side. The conventional boundary conditions of having a channel on top/bottom for treating the corresponding boundaries as walls would disregard part of the connectivity. Therefore, the average concentration from the solute profile along the y-axis is applied. This is done by calculating the average concentration in the open pores along every column (Y-axis) and adding it to the fluid voxels (if present) of that column at the upper and lower boundary.

The diffusion of the solutes through the open pore space is simulated according to the two-dimensional diffusion equation (Eq. 2) with the diffusivity D and solute concentration C.

2 $$\begin{aligned} \frac{\partial C}{\partial t} = D \left( \frac{\partial ^2 C}{\partial x^2} + \frac{\partial ^2 C}{\partial y^2} \right) \end{aligned}$$

It is calculated using the Lattice-Boltzmann method (LBM) model as presented in Prasianakis et al. (2018). The non-iterative operator splitting approach is applied to couple the LBM with geochemical reactions (Carrayrou et al. 2010). Geochemical reactions are calculated using elementary volumes. A base time-step consists of a sequence of solving the solute transport and geochemical reactions on every fluid voxel, and its duration is set during the problem dimensionalization. Heterogeneous precipitation of C-S-H is induced by a reactive zone of fluid voxels. Each fluid voxel requires at least two neighboring solid voxels to belong in the reactive zone, providing smooth solid growth. The pore space is updated at the end of every time-step by turning fluid voxels that reach 100 % precipitates into solid voxels (solidification). The accessibility of the gel pores of C-S-H for diffusion of the solutes in the RT-simulations is case-dependent.

### Surrogate Model for Geochemical Equilibria

In conventional RTMs, the mass transport solver (LBM) is coupled with a geochemical solver. At any base time-step, the solute concentrations of all voxels in the reactive zone are given as an input to the geochemical solver, which outputs the relevant values of chemical reactions (amount of precipitation of the C-S-H endmembers in this case). However, the calculations by the geochemical solvers are time-consuming due to their complex and iterative algorithms. In order to accelerate the necessary geochemical considerations, a surrogate model is coupled to the lattice Boltzmann mass transport solver instead of coupling it with the geochemical solver. The construction of the surrogate model is based on a deep neural network. It uses mathematical functions to interpolate the relation between the previously mentioned input (solute concentrations) and output (amount of precipitation) for the specific chemical system. Conceptually, it gives the same output to a given input than the geochemical solver but computationally faster. The deep neural network is trained and tested on datasets with data points generated with the geochemical solver in the concentration range that is expected to occur during the RT-simulation. This approach is known to have a very good trade-off between accuracy and speedup compared to other data-driven models and is well-established for surrogate models for geochemical equilibria (Jatnieks et al. 2016; Prasianakis et al. 2020, 2025; Demirer et al. 2023). More detailed information about the concept and state of the art of surrogate models for geochemical calculations can be found in Prasianakis et al. (2025). More specifically, the considered deep neural networks take the concentrations of $\mathrm {Ca(OH)_{2\,[aq]}}$ and $\mathrm {SiO_{2\,[aq]}}$ in mol L^− 1^ as input and provide the precipitation of the four C-S-H endmembers Tob-D, Tob-H, Jen-D and Jen-H in m^3^ L^− 1^ as output. This allows for the calculation of the volume-% of precipitates at the corresponding time-step independent of the batch reaction volume.

**Fig. 4:**
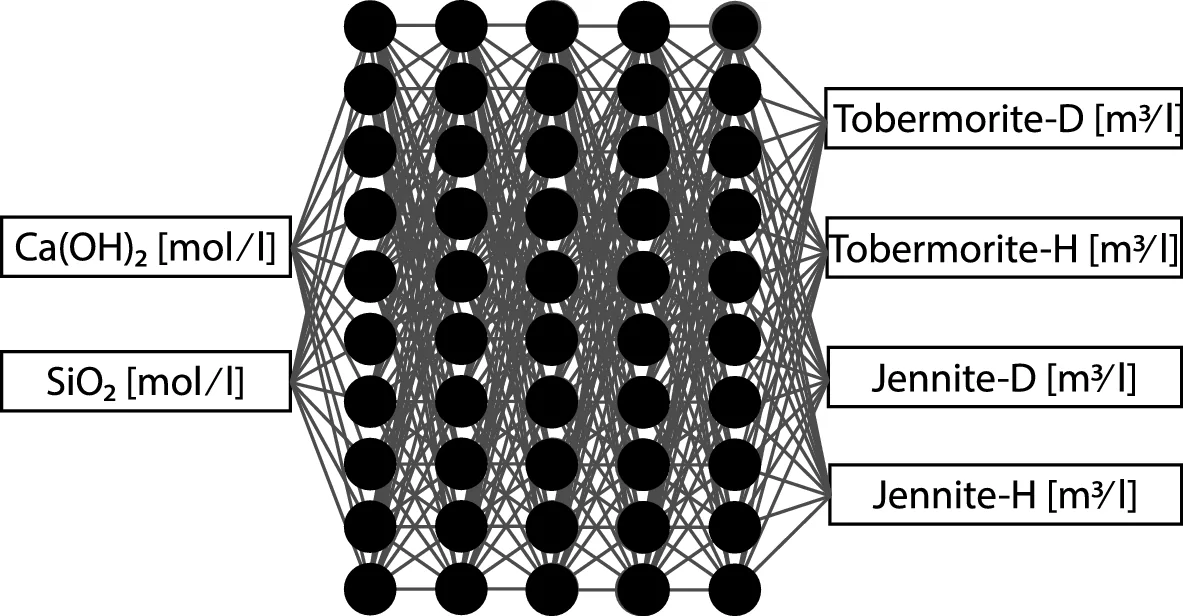
The neural network architecture used here consisting of 5 hidden layers with 10 neurons in each layer. The solute concentrations of $\mathrm {Ca(OH)_{2\,[aq]}}$ and $\mathrm {SiO_{2\,[aq]}}$ are the input, while the volume of solid precipitates of the four CSH endmembers is the output

The datasets are generated with the geochemical solver GEMS containing data points in a concentration range of 10^− 6^ to 0.02065 mol L^− 1^ for $\mathrm {Ca(OH)_{2\,[aq]}}$ and 10^− 6^ to 0.0017 mol L^− 1^ for $\mathrm {SiO_{2\,[aq]}}$. All values are converted to the logarithmic scale before the training process starts. A minimal value for the amounts of the resulting solid precipitation (precipitation threshold) is set to replace the values of zero, when a phase does not precipitate during training. This threshold facilitates the training process, since logarithmic values are used. Two neural networks were trained with a precipitation threshold of 10^− 11^ and 10^− 13^ m^3^ L^− 1^. The data-preprocessing, training and testing of the neural networks are carried out in MATLAB with the Deep Learning Toolbox. Figure 4 shows the input and outputs of the neural network models considered. In this implementation, in order to increase the accuracy of the surrogate model, the output of concentrations of aqueous species was calculated with mass balance during the RTM. This maintained a small geochemical surrogate model and ensured mass conservation during speciation and precipitation.

### Characteristic Modeling Parameters

The Ca/Si ratio of C-S-H is an important parameter, because it has an effect on the local gel porosity, thus also on the diffusivity of solutes through the pores filled by C-S-H as described in the next subchapter Effective diffusivity. The changing diffusivity of the claystone side with time influences the evolution of the microstructure, the extent of the low porosity zone and the migration of radionuclides and gas molecules through the claystone side. The relevance is described in the chapter Introduction.

#### Ca/Si Ratio of C-S-H

The Ca/Si ratio of C-S-H is calculated by dividing the number of moles of Ca (n_Ca_) by the number of moles of Si (n_Si_) per mol of C-S-H (Eq. 5). Both n_Ca_ and n_Si_ depend on the ratios of the four endmembers (X_endmember_) in C-S-H and their quantity in the corresponding endmember formula presented in Table 1 (Eq. 3 and 4).

3 $$\begin{aligned} n_{\textrm{Si}}= & \left( X_{\mathrm {Tob\text {-}H}} + X_{\mathrm {Jen\text {-}H}} \right) + \frac{2}{3} \left( X_{\mathrm {Tob\text {-}D}} + X_{\mathrm {Jen\text {-}D}} \right) \end{aligned}$$

4 $$\begin{aligned} n_{\textrm{Ca}}= & \frac{2}{3} X_{\mathrm {Tob\text {-}H}} + \frac{4}{3} X_{\mathrm {Jen\text {-}H}} + \frac{5}{6} X_{\mathrm {Tob\text {-}D}} + \frac{3}{2} X_{\mathrm {Jen\text {-}D}} \end{aligned}$$

5 $$\begin{aligned} \mathrm {Ca/Si}_{\mathrm {(C\text {-}S\text {-}H)}}= & \frac{n_{\textrm{Ca}}}{n_{\textrm{Si}}} \end{aligned}$$

#### Effective Diffusivity

The effective diffusivity can be measured numerically at any stage of the evolution of the system by extracting the evolved pore structure including the new C-S-H precipitates. It is measured with diffusion simulations computed by the LBM (as described in subchapter Reactive Transport Model but without coupling of geochemical reactions) by applying a concentration gradient along the boundaries of the porous medium in the direction of interest, and measuring the resulting mass flux at steady state. The diffusivity through the gel pores, which are accessible for mass transport, is taken into account in all diffusion simulations. A sub-grid model is considered for the gel pores because the exact geometrical features of the gel pores are not known, and because their length scale (nanometer scale) is smaller than the considered computational voxel size (micrometer scale). A local diffusion coefficient D_local_ is assigned to every voxel depending on the volume of C-S-H precipitates and its gel porosity, which depends on the state of the solid solution according to Eq. 14. The first step in calculating the gel porosity consists of obtaining the ratio between water (H) and silica (S) in the gel pores H/S_gel_. This is calculated by subtracting the water-to-silica ratio in the interlayer H/S_il_ from the ratio in the bulk H/S_bulk_. The calculations of H/S_il_ (Eq. 6) and H/S_gel_ (Eq. 8) follow the model of (Kulik 2011).

6 $$\begin{aligned} \mathrm {H/S}_{\textrm{il}}= & \frac{7}{6} \left( X_{\mathrm {Tob\text {-}H}} + X_{\mathrm {Jen\text {-}H}} \right) + \frac{8}{6} \left( X_{\mathrm {Tob\text {-}D}} + X_{\mathrm {Jen\text {-}D}} \right) \end{aligned}$$

7 $$\begin{aligned} \mathrm {H/S}_{\textrm{bulk}}= & \frac{9}{6} X_{\mathrm {Tob\text {-}H}} + \frac{13}{6} X_{\mathrm {Jen\text {-}H}} + \frac{11}{4} X_{\mathrm {Tob\text {-}D}} + \frac{15}{4} X_{\mathrm {Jen\text {-}D}} \end{aligned}$$

8 $$\begin{aligned} \mathrm {H/S}_{\textrm{gel}}= & \mathrm {H/S}_{\textrm{bulk}} - \mathrm {H/S}_{\textrm{il}} \end{aligned}$$

The next step consists of the calculation of the weight fraction of the water in the gel pores. This is done by dividing the weight of water in C-S-H by the molar weight of C-S-H (M_C-S-H_) (Eq. 10). M_C-S-H_ depends on the endmember ratio and their corresponding molar weight (Eq. 8). The weight of water in C-S-H is calculated by multiplying the molar weight of water ($M_{\mathrm {H_2O}}$ = 18 g mol^-1^) by the moles of water that occur in the gel pores. The latter is calculated by multiplying H/S_gel_ with $n_{\mathrm {SiO_2}}$.

9 $$\begin{aligned} M_{\mathrm {C\text {-}S\text {-}H}}= & 169 \cdot X_{\mathrm {Jen\text {-}D}} + 174 \cdot X_{\mathrm {Jen\text {-}H}} + 120 \cdot X_{\mathrm {Tob\text {-}D}} + 124 \cdot X_{\mathrm {Tob\text {-}H}} \end{aligned}$$

10 $$\begin{aligned} \mathrm {H_2O}_{\textrm{gel}}~[\mathrm {weight\text {-}fraction}]= & \frac{M_{\mathrm {H_2O}} \cdot n_{\mathrm {H_2O,gel}}}{M_{\mathrm {C\text {-}S\text {-}H}}} \end{aligned}$$

The volume fraction of water in the gel pores is obtained by multiplying the weight fraction of water in the gel pores by the density of C-S-H (Eq. 12). The density of C-S-H depends on the endmember ratio and their corresponding densities in g cm^− 3^ from Table 1 (Eq. 11).

11 $$\begin{aligned} \rho _{\mathrm {C\text {-}S\text {-}H}}= & 2.1 \cdot X_{\mathrm {Jen\text {-}D}} + 2.3 \cdot X_{\mathrm {Jen\text {-}H}} + 2.5 \cdot X_{\mathrm {Tob\text {-}D}} + 2.25 \cdot X_{\mathrm {Tob\text {-}H}} \end{aligned}$$

12 $$\begin{aligned} \mathrm {H_2O}_{\textrm{gel}}~[\mathrm {voume\text {-}fraction}]= & \mathrm {H_2O}_{\textrm{gel}}~[\mathrm {wt\text {-}fraction}] \cdot \rho _{\mathrm {C\text {-}S\text {-}H}} \end{aligned}$$

The gel porosity ($\varepsilon _{\textrm{gel}}$) directly corresponds to the volume fraction of gel pore water in the C-S-H (H_2_O_gel_) (Eq. 13).

13 $$\begin{aligned} \varepsilon _{\textrm{gel}} = \mathrm {H_2O}_{\textrm{gel}}~[\mathrm {volume\text {-}fraction}] \end{aligned}$$

The diffusion coefficient of C-S-H with the gel pores D_gel_ is related to the gel porosity via Archie’s Law with a cementation factor of 2 and the diffusion coefficient in pure water (D_0_), which is assigned to 1 (Eq. 14). In the literature, no cementation factor specifically for C-S-H was found. However, Luraschi et al. (2020) suggested a cementation factor of 2 for anions and 4.4 for uncharged molecules in the low porosity zone of the cement–claystone interface due to the precipitation of mainly C-S-H. The cementation factor of 2 was chosen here.

14 $$\begin{aligned} D_{\text {gel}} = \varepsilon _{\text {gel}}^{2} \cdot D_{0} \end{aligned}$$

The final local diffusion coefficient D_local_ requires consideration of the ratio between the C-S-H precipitates and the fluid in the corresponding voxel. It is simply calculated by adding the ratio of C-S-H (X_C-S-H_) multiplied by the diffusion coefficient through the C-S-H with the ratio of fluid (1-X_C-S-H_) multiplied by the diffusion coefficient in pure water (Eq. 15). Consequently, D_local_ is equivalent to D_0_ in a fluid voxel without any C-S-H and to D_gel_ when it is completely filled with C-S-H.

15 $$\begin{aligned} D_{\text {local}} = (1 - X_{\text {C-S-H}}) \cdot D_0 + X_{\text {C-S-H}} \cdot D_{\text {gel}} \end{aligned}$$

## Adaptive Time-Stepping

Realistic pore-scale RTM require fine discretization of the simulation domain and short time scale to capture the temporal evolution of the system or to reach a steady-state condition, leading to the high computational costs. For example, a fluid voxel in the reactive zone of the simulation in this paper obtains around 10^− 8^ volume-% C-S-H precipitates per time-step, thus requiring 10^8^ time-steps for the precipitates to occupy a volume equal to one voxel. At the same time, the diffusion and precipitation fields require around 10^5^ time-steps to reach the first stationary state. This clear separation of timescales on the micrometer scale between relatively fast diffusion, fast precipitation reaction, and slow solid growth (beyond the size that becomes significant) highlights the multiscale nature of the problem.

### Reactive Transport States

**State I: Initial Transient State**

The simulation starts with an initial concentration field on the bulk and on the fixed concentration at the opposite boundary sides to establish a geochemical gradient. A strong solute flux is generated as a response of the system to the geochemical gradient. In an initial transient state, the diffusion field (while being affected by the precipitation field) dynamically evolves toward a stationary state to balance the chemical gradient. This phase occurs only at the beginning of the simulation. The extent of the dynamically changing diffusion field in this phase is generally higher than in later transient stages (State IV) and depends on the initial concentration, geochemical gradient, complexity of the microstructure and simulation domain size.

**State II: Temporary Onset of Quasi-Steady State (TOSS)**

In the case of slow kinetics, the diffusion field can reach a stationary state before any significant changes in the microstructure from solid growth. This stabilizes the precipitation field, which will also reach a stationary state resulting in constant local solid growth conditions across the domain. This state is referred to as the Temporary Onset of quasi-Steady State, shortly TOSS. Downscaled to one voxel in the reactive zone, it is characterized by constant values of solute flux (computed by the mass transport solver), precipitation rate (calculated by the surrogate model), and solid growth (added number of precipitates occupying the voxel volume).

**State III: Disturbance of the TOSS**

The continued solid growth during the TOSS in State II leads to the evolution of the microstructure and significant solid growth is necessary to disturb the diffusion field (and therefore also disturbing the precipitation field). In this RTM setup, such a triggering event is the solidification of fluid voxels. The extent of solidifying fluid voxels necessary to disturb the TOSS depends on the impact of the solidified voxels on the solute transport pathway, the total number of voxels in the simulation domain and the complexity of the pore space.

**State IV: Rearranging Transient state**

After the disruption of the TOSS, the diffusion field dynamically evolves toward the next TOSS. This state is analogous to State I, with the difference of weaker dynamics in the diffusion and precipitation fields in this state and has typically shorter duration. The solidification of voxels that trigger the disruption of the TOSS change the diffusion and precipitation field less strongly than the balancing of the chemical gradient in State I.

**Fig. 5:**
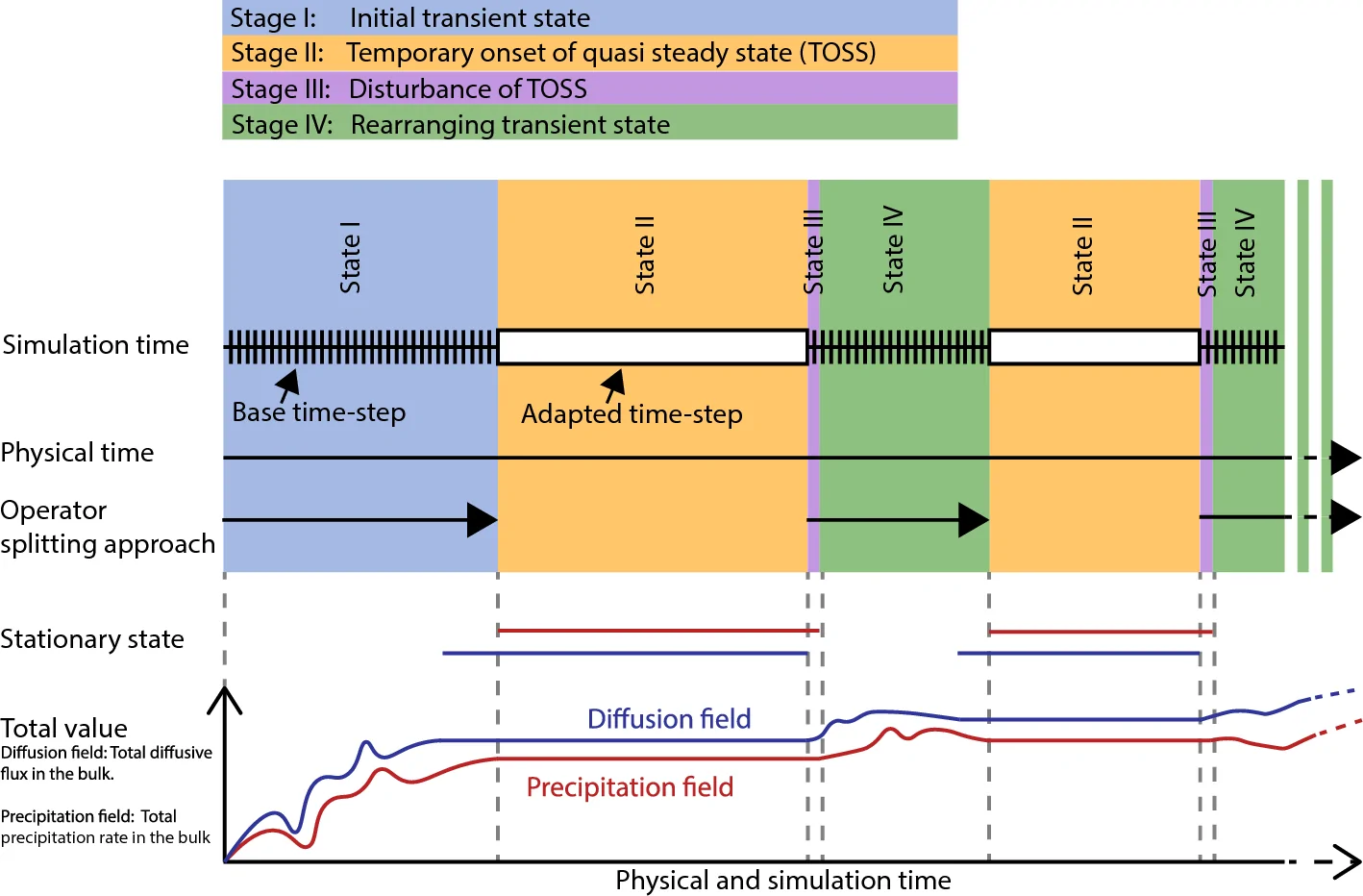
Illustrative scenario of an evolving reactive transport simulation. The initial transient state (State I, blue) is characterized by a dynamically evolving diffusion field and precipitation field, which is simulated with base time-steps. The temporary occurrence of quasi-steady state (TOSS, State II, orange) begins when the diffusion field and precipitation field both reach a stationary state. This step is simulated with the adapted time-step. It ends with the onset of the disturbance of the TOSS (State III, purple), when the diffusion field loosens from the stationary state. This initiates the rearranging transient state (State IV, green) with a dynamically evolving diffusion and precipitation field (less than State I). The system can reach a stationary state again and a further TOSS begins. The cycle of State II to IV continues until the simulation is completed. Note, that the physical time during TOSS is not simulated by the operator splitting approach with the base time-steps but with an adapted time-step, which leads to the computational acceleration of the adaptive time-stepping algorithm

State II to IV are observed several times during the simulations. The significance of this system state evolution cycle comes from the fact that the simulation of the diffusion field with the mass transport solver and the precipitation field with the surrogate model is redundant during the TOSS (State II) because they are in a stationary state. This observation is key in taking advantage of this clear separation of phenomena’ time scales (Fig. 5).

The adaptive time-stepping algorithm is described in the following section. In summary, it monitors the evolution of the diffusion field and the precipitation field to detect possible TOSS (State II). When detected, it dynamically adapts the time-step by simulating only the solid growth while considering the diffusion field and precipitation field in a stationary state. The time-step of the solid growth is adapted to a clearly defined limit that directly sets off to the disruption of the TOSS (State III). Consequently, simulations of the diffusion field and precipitation field during the TOSS (State II) are essentially skipped. The accuracy and efficiency of the algorithm are in principle controlled by three parameters, namely the pre-activation phase, TOSS threshold and screening window factor.

#### Adaptive Time-Stepping Algorithm

**Fig. 6:**
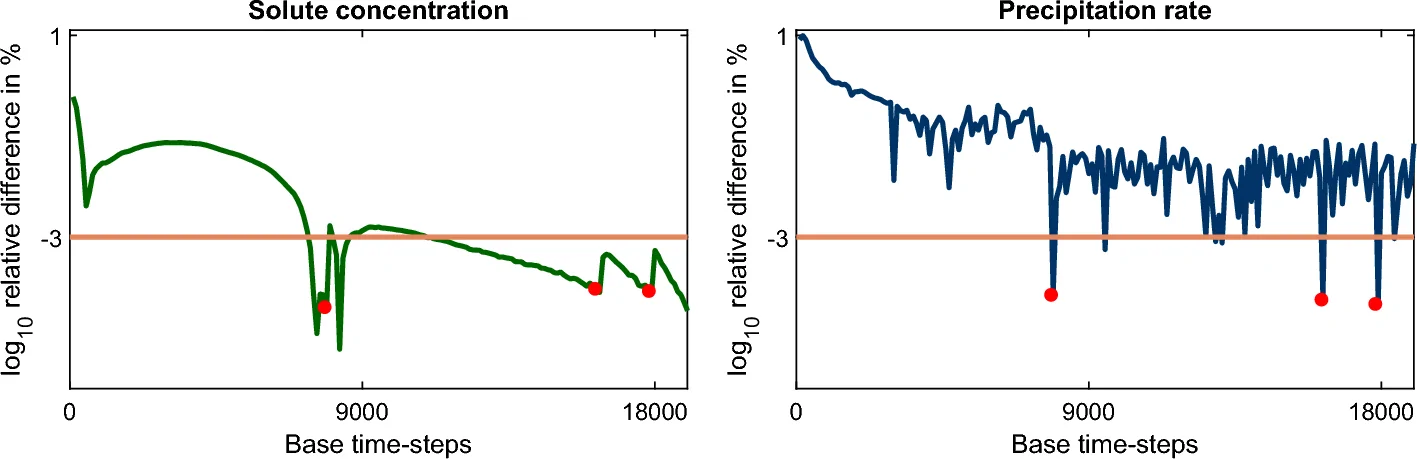
Evolution of the absolute relative differences in % of the change of solute concentration (left) and precipitation rate (right). An adapted time-step is activated (red dot) when both reach values below the TOSS threshold ($10^{-3}$ $\frac{\%}{50\ \text {base time-steps}}$ in this example)

As described before, State I can be characterized by strong dynamics in the evolving diffusion field. In practice, it is shown that it is slightly more efficient to have a stricter control of detecting the first TOSS (during State I). This is handled by defining a range of base time-steps at the beginning of the simulation before the algorithm engages, called the pre-activation phase. After the simulation exceeds this pre-activation phase, the algorithm starts to monitor the state of the diffusion field and precipitation field to detect the potential TOSS. The monitoring is carried out by calculating the relative percentage difference between the total precipitation rate (P) (Eq. 16) and the diffusive flux (J) (Eq. 17) with time. For the calculation of the relative difference of the fields, an integral of the total computational domain is considered instead of single monitoring points, since different parts of the pore space might converge toward the stationary state at different rates. Such models, depending on the training accuracy, might introduce infinitesimal fluctuations/oscillations which can propagate to the calculation of the local precipitation rates and diffusive fluxes. Therefore, a time-averaging scheme (over a few base time-steps) is considered for the identification of the relaxed states. In the system of interest, the average ($\overline{P}$ and $\overline{J}$) of the last 20 base time-steps (n) was compared to the average of the previous 30 to 50 base time-steps to calculate the relative percentage difference in the concentration field and the reaction rate. A TOSS is detected when the inequalities of Eq. 16 and 17 are satisfied. This essentially identifies when both the diffusion field and precipitation field reach a stationary state. At this point, an adapted time-step is initiated. An example of the monitoring is shown in Fig. 6)

16 $$\begin{aligned} & \left( \frac{ \overline{P}_{[n-20,\, n]} - \overline{P}_{[n-50,\, n-30]} }{ \overline{P}_{[n-50,\, n-30]} } \right) \cdot 100\% < \text {TOSS threshold} \end{aligned}$$

17 $$\begin{aligned} & \left( \frac{ \overline{J}_{[n-20,\, n]} - \overline{J}_{[n-50,\, n-30]} }{ \overline{J}_{[n-50,\, n-30]} } \right) \cdot 100\% < \text {TOSS threshold} \end{aligned}$$

**Step I: Determination of the Prime Voxel**

Step I of an adapted time-step considers the determination of the voxel that will solidify next (“prime voxel”) if the simulation continues in its stationary state. It depends on the local precipitation rate and the degree of precipitates that each voxels contains. This allows us to extrapolate and identify the expected time needed to completely fill the voxel with precipitates. Equation 18 is applied to every voxel in the simulation domain where it calculates and registers the theoretically required base time-steps T_Solidification_ needed to solidify the respective voxel when constant reaction rate conditions are considered with X_C-S-H_ being the ratio of C-S-H in % inside the respective voxel and P the precipitation rate in $\frac{\%}{\text {base time-step}}$). The voxel with the lowest value of T_Solidification_ is defined as the prime voxel.

18 $$\begin{aligned} T_{\text {Solidification}} = \frac{100\% - X_{\text {C-S-H}}}{P} \end{aligned}$$

**Step II: Screening Window to Determine Affiliated Voxels**

This step considers the prevention of having several sequential adapted time-steps for each solidifying voxel and to increase the benefits of a single adapted time-step. The solidification of a single voxel in a porous medium is expected to not have a significant effect on the diffusion field and precipitation field. In other words, the stationary state criteria are not expected to be very sensitive to the changes resulting from a single voxel. This is a very crucial consideration for large geometries consisting of millions to billions of voxels. The algorithm detects additional voxels (“affiliated voxels”) that would solidify in a close span of base time-steps after the prime voxel. This is achieved by using a screening window of a certain number of base time-steps on top of T_Solidification_ from the prime voxel to check for potential voxels that would solidify during this time span. The screening window factor has a value of 1 or greater and multiplies with the value of T_Solidification_ of the prime voxel determined from Eq. 18 in Step I. Every voxel that satisfies the inequality of Eq. 19, thus having a value of T_Solidification_ inside the screening window, is defined as an affiliated voxel, which will be part of the solidifying voxels within a single step.

19 $$\begin{aligned} T_{\text {Solidification, prime voxel}} \cdot \text {Screening window factor} < T_{\text {Solidification}} \end{aligned}$$

**Step III: Determination and Application of the Adapted Time-Step**

The adapted time-step per construction should solidify the prime voxel and the affiliated voxels in a single step. Therefore, the highest value of T_Solidification_ from Eq. 18 inherited by the prime voxel or affiliated voxels determines the extent of the adapted time-step. All precipitating voxels in the reactive zone then grow with their respective growth rate by multiplying the adapted time-step with the local precipitation rate on the respective voxel. It is important to mention that the adapted time-step focuses on the consistent acceleration of solid growth.

**Step IV: Consistent Treatment for Heterogeneous Precipitation**

**Fig. 7:**
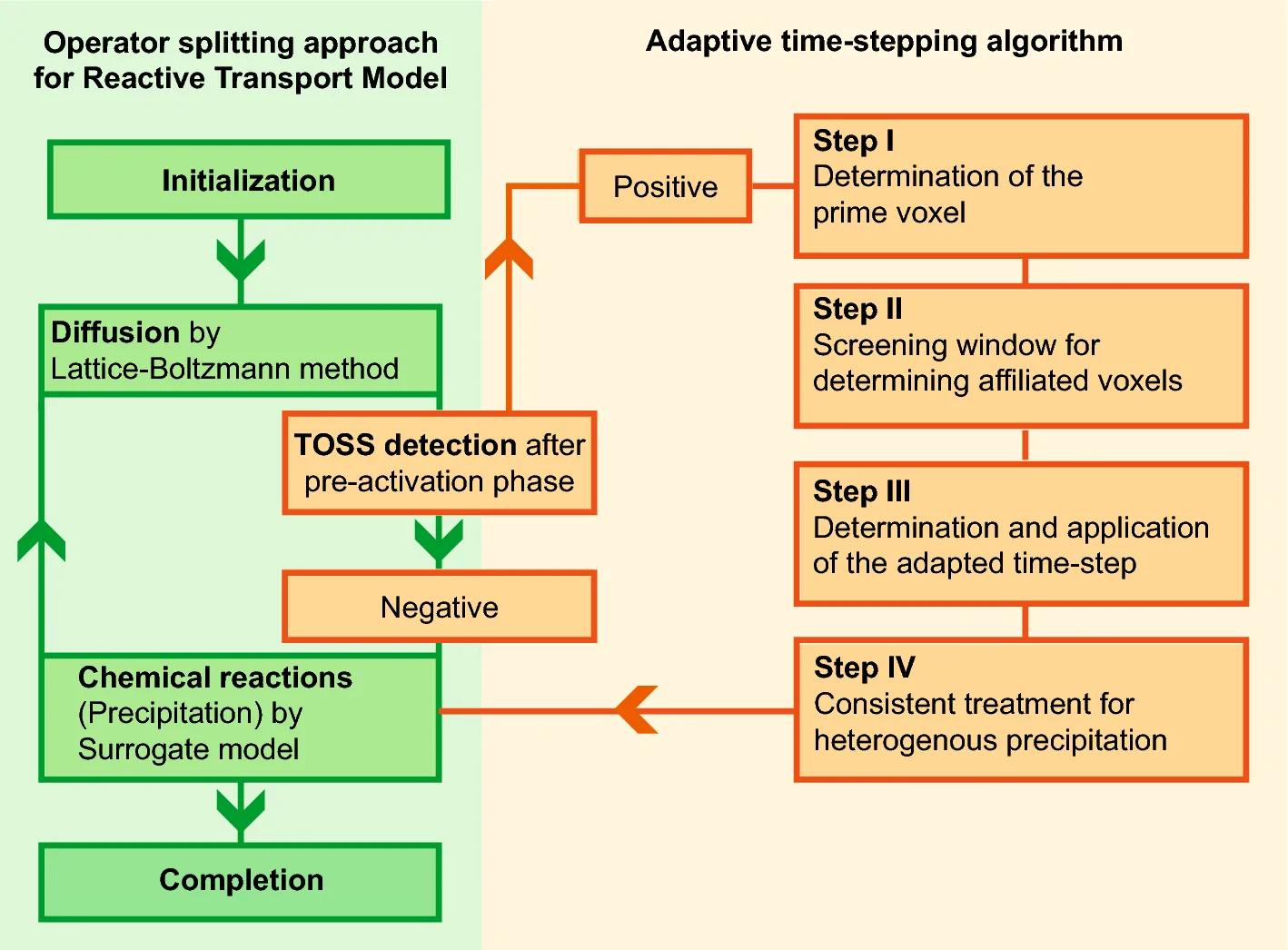
Flow diagram of the RTM (green) and the adaptive time-stepping algorithm (orange) with the four steps which are executed when a TOSS is detected

As a consequence of applying an adapted time-step that potentially solidifies several voxels at once, some of the solidifying voxels can reach more than 100 volume-% of precipitates if no special care is taken. Moreover, the voxels which reach first the full solid occupancy and are in contact with the fluid should extend the reactive zone by adding and activating the neighboring fluid voxels (as soon as there are two neighboring solid voxels which are exposed to fluid voxels). Such voxels are added to the reactive zone after the acceleration step (after the time span specified by the adapted time step), and they have to be treated in a consistent way in order to not miss the corresponding precipitates. The algorithm is therefore designed to determine the number of base time-steps T_Excess_ that led to the potential overshooting solid growth on the prime voxel and affiliated voxels. This is done by dividing the excess volume-% of precipitates (if it is more than 100%) by the local precipitation rate in the respective voxel. T_Excess_ is then distributed to the potential neighboring fluid voxels, which are newly added to the reactive zone. From that point on, the simulation continues for T_Trigger_ number of base time-steps with the operator splitting approach but without monitoring for TOSS. Implementing T_Trigger_ is necessary to obtain possible precipitation rates in voxels that are recently added to the reactive zone. T_Trigger_ is set to 100 base time-steps in this system of interest. After T_Trigger_ base time-steps, the local precipitation rates are multiplied by T_Excess_ time-steps of the corresponding voxel. This completes an adapted time-step, and the simulation continues with the operator splitting approach until the system reaches the conditions where the next TOSS is detected (Fig. 7).

## Results

In this section, the adaptive time-stepping algorithm for RT-simulations is scrutinized for its accuracy and efficiency. RT-simulations with the adaptive time-stepping algorithm are performed for three systems which differ in domain size, microstructure, accessibility of gel pores for mass transport (Table 2), boundary and initial conditions.

**Table 2:** Setup of the three cases used to test the adaptive time-stepping algorithm

	Case I	Case II	Case III
Simulation domain size in μm	2 x 0.86	10 x 6.25	10 x 6.25
Simulation domain size in voxels	114 x 50	241 x 151	241 x 151
Resolution in μm	0.018	0.04	0.04
Initial porosity	0.34	0.27	0.27
Gel pores during RT-simulation	closed	closed	open
Gel pores during diffusion simulation	open	open	open

The three adaptive time-stepping parameters are tested by performing RT-simulations with different values of the respective parameter, while the other two parameters are set to values that previously resulted in good accuracy to minimize their influence on the outcome. Detailed information on adaptive time-stepping parameters, the calculation of the relative deviation, and acceleration factor to measure the efficiency and accuracy of the algorithm can be found in Supplementary information.

### Case I: Small Simulation Domain

**Fig. 8:**
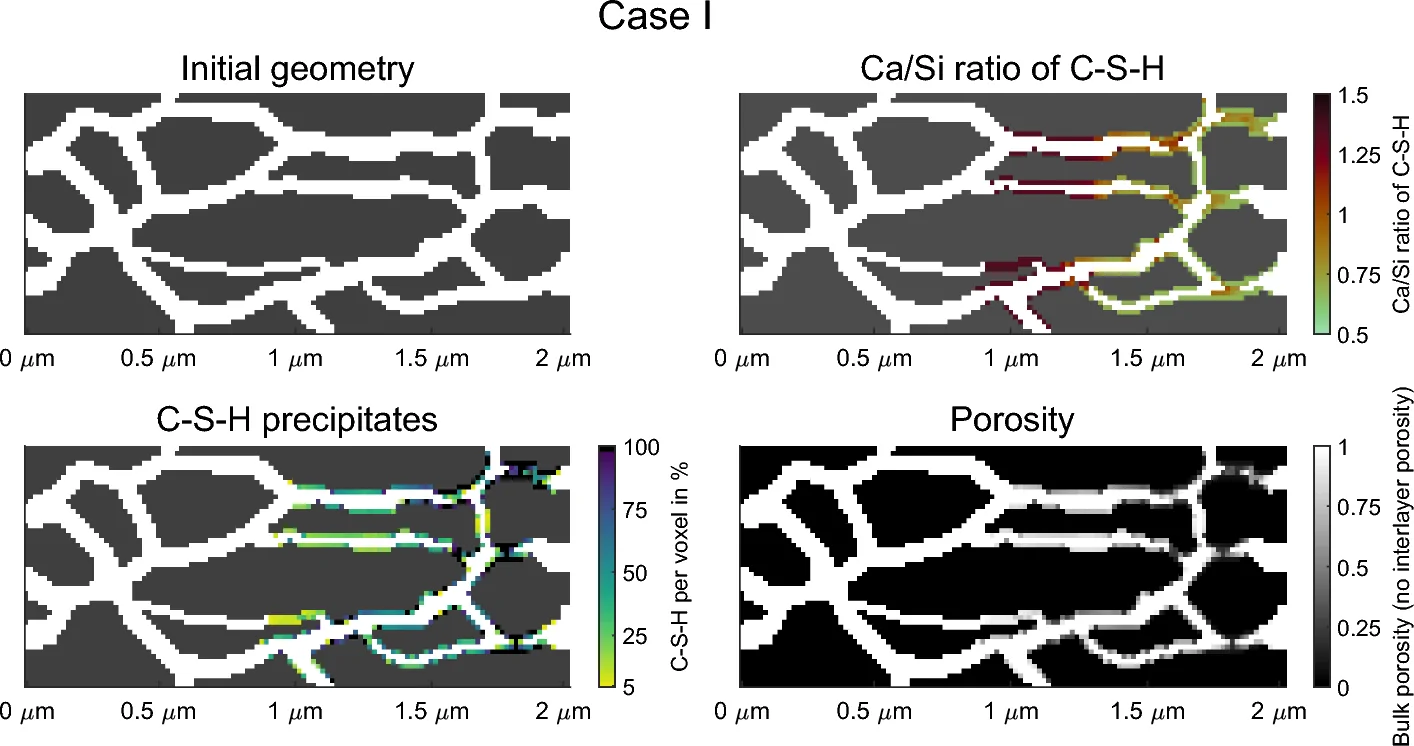
Initial geometry of the porous media for Case I with the 2 x 0.86 μm simulation domain (top, left). Simulation results at a time close to clogging conditions with information about Ca/Si ratio of C-S-H (top, right, only for voxels with more than 5% C-S-H precipitates), C-S-H precipitates (bottom, left) and porosity (bottom, right). The results stem from the simulation without the implementation of the adaptive time-stepping algorithm

The first benchmark is conducted in a relatively small simulation domain that represents the pore space of a claystone with irregularly shaped clay minerals (Fig. 8, top left) and the initial and boundary conditions in Table 3. A neural network-based surrogate model with a precipitation threshold of 10^− 11^ m^3^ L^− 1^ is used for these RT-simulations because they are characterized by a precipitation of around 10^− 10^ m^3^ L^− 1^ per base time-step and voxel. The neural network has an architecture of 5 hidden layers with 10 neurons in each layer. It accelerates the RT-simulations by 41 times (excluding the effect of the adaptive time-stepping).

**Table 3:** Boundary and initial conditions for Case I

	Left boundary	Right boundary	Initial bulk
$\mathrm {Ca(OH)_{2\,[aq]}}$	Fixed at 2.5 mmol L^-1^	Neumann (zero rate of change)	0.1 mmol L^-1^
$\mathrm {SiO_{2\,[aq]}}$	Fixed at 0.1 mmol L^-1^	Fixed at 1.7 mmol L^-1^	1.7 mmol L^-1^

Figure 8 shows the results of the simulation for Case I (while applying the geochemical surrogate model but not the adaptive time-stepping algorithm) at clogging (final state) for the most important parameters which are the local porosity, C-S-H precipitates and Ca/Si ratio of C-S-H within the pores. The calculation and relevance of those parameters are described in characteristic modeling parameters. It is apparent that the C-S-H precipitates at the time of clogging are mostly distributed on the right side of the simulation domain (2.5% on the left half, 97.5% in the right half). The $\mathrm {Ca(OH)_{2\,[aq]}}$ are consumed by the C-S-H precipitation at the right side before enough solutes can diffuse further into the simulation domain to ensure supersaturation. The Ca/Si ratio of the C-S-H has values of 0.67 to 0.75 close to the right boundary and increases to values of 1.4 toward the middle (Fig. 8, top right). This difference is the result of the precipitates having a high ratio of Tob-H and an intermediate of Tob-D on the right side to an increasing ratio of Jen-H toward the middle. In general, there was no significant precipitation of Jen-D. The precipitation of C-S-H alters the structure of the pore space by decreasing the local porosity (Fig. 8, bottom left). The solidified voxels on the right side of the pore space that led to clogging have a local porosity of 0.11 to 0.2 when the gel pores of C-S-H are considered.

In the following three subchapters, the adaptive time-stepping algorithm is enabled and tested against the result of the reference simulation (no adaptive time-stepping).

#### Adaptive Time-Stepping: Effect of TOSS Threshold

**Fig. 9:**
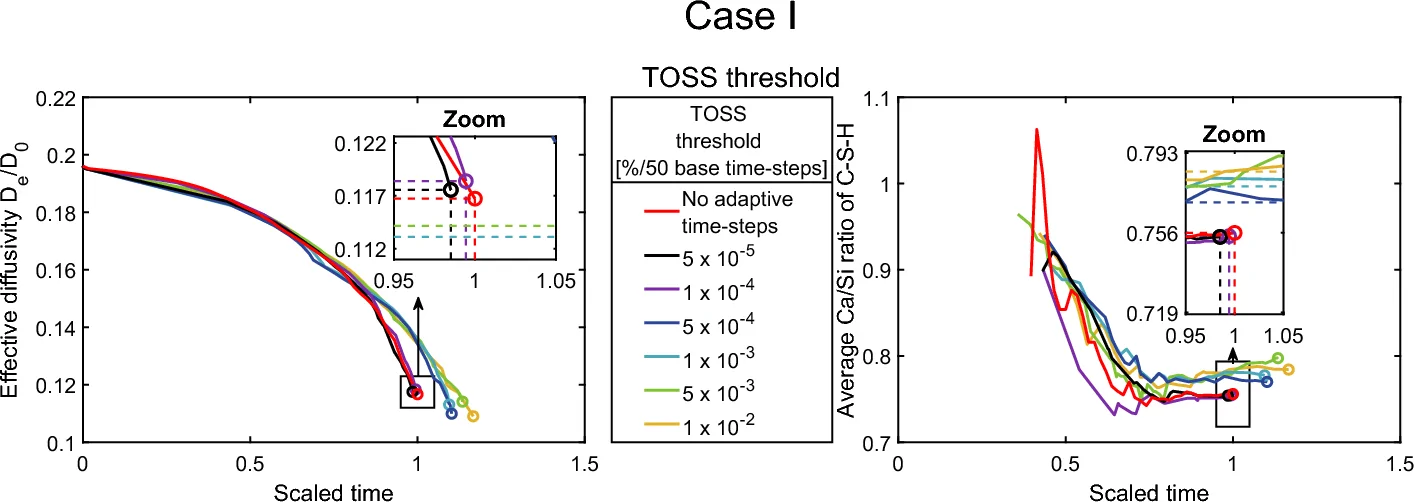
Time evolution of the effective diffusivity (left) and Ca/Si ratio of C-S-H (right) for all simulations of varying the TOSS threshold in Case I. Lower TOSS thresholds (black and purple curves) result in system evolutions which are in excellent agreement with the reference simulation (red curve). Higher TOSS thresholds (blue, green and yellow curves) result in larger deviation in the time evolution

The time evolution of the effective diffusivity and average Ca/Si ratio of C-S-H for all simulations from the set of varying the TOSS thresholds for Case I and the reference simulation without the adaptive time-stepping algorithm (red) are displayed in Fig. 9. The time evolution of the effective diffusivity and the Ca/Si ratio of C-S-H for the simulations with a TOSS threshold of $5 \cdot 10^{-5}$ and $10^{-4}$ $\frac{\%}{50\ \text {base time-steps}}$ agree very closely with the reference simulation. For the time evolution of the effective diffusivity, higher TOSS thresholds have a close resemblance up to a scaled time of 0.6. Afterward, the deviation starts to become significant.

For the time evolution of the Ca/Si ratio in C-S-H, higher TOSS thresholds lead to an overprediction which is more pronounced with time. The higher TOSS thresholds also lead to an overprediction of the time at clogging (end of the simulation), which further contributes to the different time evolutions, the underestimation of the effective diffusivity and the overestimation of the Ca/Si ratio of C-S-H. The reference simulation has a bump of the Ca/Si ratio of C-S-H at a scaled time of 0.45 (Fig. 9, right), which is not captured in the other simulations. This bump comes from the first two solidifying voxels. The simulations with the adaptive time-stepping algorithm solidify several voxels at the first temporal quasi-steady state which average out the first two voxels in the displayed time evolution. It should be noted that the pores have diameters of four to eight voxels. Therefore, only a few voxels must be solidified to clog a pore throat. In these simulations (Case I), the gel pores are not accessible for mass transport during the reactive transport and clogging of the pores will stop the counter-diffusion of the solutes and no precipitation will occur after clogging. Consequently, the period of solidifying the first voxels makes up a large portion (from 0 to 0.45 scaled time) of the total time evolution.

**Fig. 10:**
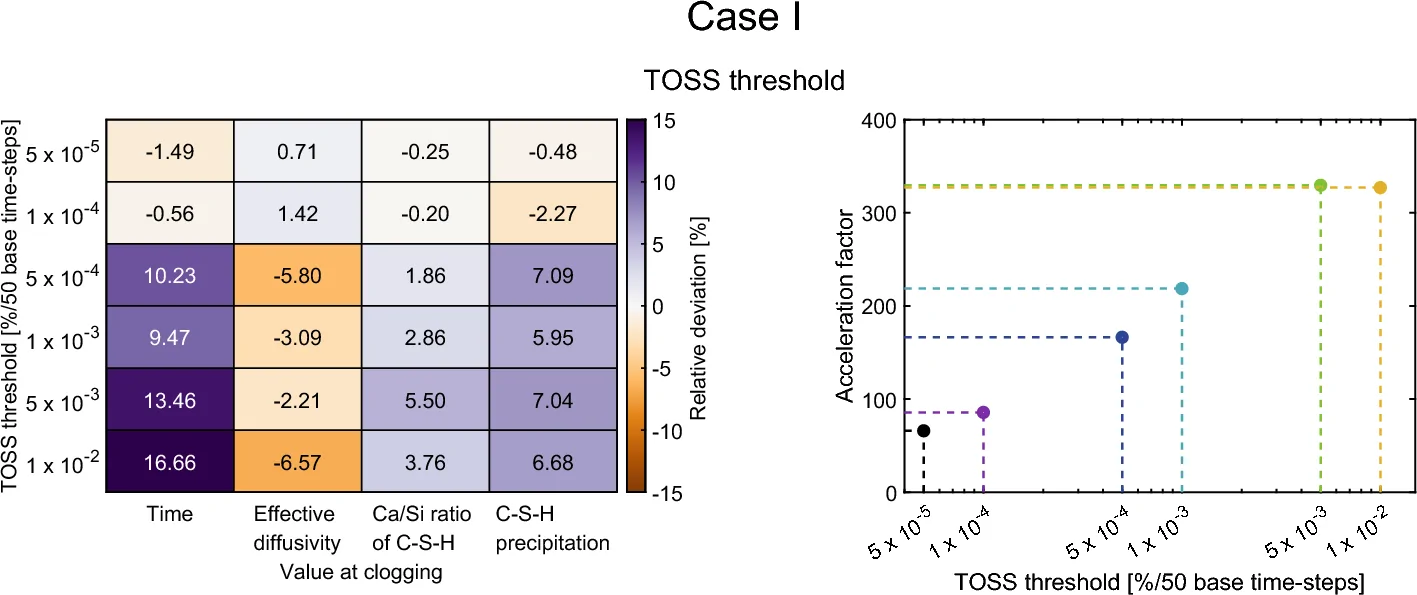
Heatmap with the relative deviations in % compared to the reference simulation for the simulation set of varying the TOSS threshold for relevant parameters at clogging (left) and plot for the acceleration factors (right) for Case I. Lower TOSS thresholds lead to higher acceleration factors, but also higher deviations from the reference simulation

The relative deviations for relevant parameters at clogging between the simulations with different TOSS thresholds and the reference simulation are displayed in a heatmap (Fig. 10, left). The TOSS threshold of $5 \cdot 10^{-5}$ and $10^{-4}$ $\frac{\%}{50\ \text {base time-steps}}$ has absolute relative deviations below 1.5% and 2.3%, respectively. The accuracy decreases with higher TOSS thresholds values. This effect is most prominent for the time with absolute relative deviations above 10% for the TOSS threshold of $5 \cdot 10^{-4}$ $\frac{\%}{50\ \text {base time-steps}}$, up to 16.7% for $10^{-2}$ $\frac{\%}{50\ \text {base time-steps}}$. It is less prominent for the Ca/Si ratio of C-S-H with absolute relative deviations below 6% for all TOSS thresholds. The trend of decreasing accuracy is less pronounced for the effective diffusivity and C-S-H precipitation with absolute relative deviations up to 7%.

Generally, lower TOSS thresholds lead to higher acceleration factors. At the same time, accuracy decreases with lower TOSS thresholds. The selection of the TOSS threshold allows us to balance the gains in computational time with respect to the simulation accuracy depending on the available computational resources and the accuracy acceptance criteria (fidelity of the simulation). For example, the simulation with the TOSS threshold of $5 \cdot 10^{-5}$ $\frac{\%}{50\ \text {base time-steps}}$ has a total acceleration factor of 60 times (Fig. 10, right). Higher TOSS threshold values lead to a steady increase in acceleration factors to 330 for the TOSS threshold of $10^{-2}$ $\frac{\%}{50\ \text {base time-steps}}$. The TOSS threshold of $10^{-4}$ $\frac{\%}{50\ \text {base time-steps}}$ that received absolute relative deviations below 2.5% led to an acceleration of 90 times.

#### Effect of Pre-activation Phase

The results of the simulations of the set of varying the pre-activation phase for Case I are presented here with more detailed plots in Supplementary information. This phase is essentially simulating the initial transient phenomena that exist due to the setup of the boundary conditions of the simulations and is directly related to the initialization of the simulation setup. The pre-activation phase can make up a significant portion of the total simulation duration of around 1’000’000 base time-steps with the implementation of the adaptive time-stepping algorithm. Analogous to the TOSS threshold, the selection of the pre-activation phase allows one to balance the gains in computational time with respect to the simulation accuracy. For example, the simulations with pre-activation phases of 300’000 and 200’000 base time-steps have absolute relative deviations below 2% and 2.3%, respectively, for all four parameters (time, effective diffusivity, Ca/Si ratio of C-S-H, total precipitation of C-S-H) at clogging. The simulations with pre-activation phases of 100’000 and 0 base time-steps have absolute relative deviations between 3% and 8%, respectively. They are higher for the time and effective diffusivity (up to 8%), intermediate for the precipitation of C-S-H (up to 5%) and low for the Ca/Si ratio of C-S-H (up to 3%). The acceleration factor slightly increased from 89 with a pre-activation phase of 300’000 to 100 with a pre-activation phase of 0 base time-steps. This suggests that a long pre-activation phase does not have a significant effect on the acceleration factor but it should also not be neglected.

#### Effect of the Screening Window

The results of the simulations of the set of varying the screening window factor for Case I are presented here with more detailed plots in Supplementary information. There is no significant trend apparent between the accuracy and screening window factors. All simulations have absolute relative deviations below 3.1% for the C-S-H precipitation and effective diffusivity and below 1.6% for the time and Ca/Si ratio of C-S-H at clogging. The highest tested screening window factor of 2.5 leads to an acceleration factor of 150, compared to the acceleration factor of 95 with a screening window factor of 1. The effect of the screening window on the required number of adapted time-steps and the number of solidified voxels per adapted time-step is shown in Table 4. Higher gains are expected when large simulation domains are considered.

**Table 4:** The effect of the screening window factors in Case I on the number of adapted time-steps required to complete the RT-simulations and the average number of solidified voxels per adapted time-step

Screening window factor [-]	Number of adapted time-steps	Solidified voxels per adapted time-step
1.00	96	1.0
1.11	91	1.1
1.25	77	1.4
1.43	65	1.6
1.67	33	3.2
2.50	14	8.4

### Case II: Large Simulation Domain

Simulations were also performed on a second case characterized by a larger simulation domain and different initial and boundary conditions (Table 5). A neural network-based surrogate model with a precipitation threshold of 10^− 13^ m^3^ L^− 1^ is used for these RT-simulations because they are characterized by a precipitation of around 10^− 12^ m^3^ L^− 1^ per base time-step and voxel. The neural network has an architecture of 6 hidden layers with 8 neurons in each layer. It accelerates the RT-simulations by 54 times (excluding the effect of the adaptive time-stepping). RT-simulations without the adaptive time-stepping algorithm were not feasible (computationally too expensive). Therefore, the simulation with the least strict tested adaptive time-stepping parameter is chosen as the reference simulation.

**Table 5:** Boundary and initial conditions for Case II

	Left boundary	Right boundary	Initial bulk
$\mathrm {Ca(OH)_{2\,[aq]}}$	Fixed at 1.1 mmol L^− 1^	Fixed at 0.81 mmol L^− 1^	0.81 mmol L^− 1^
$\mathrm {SiO_{2\,[aq]}}$	Fixed at 0.64 mmol L^− 1^	Fixed at 1.1 mmol L^− 1^	0.64 mmol L^− 1^

**Fig. 11:**
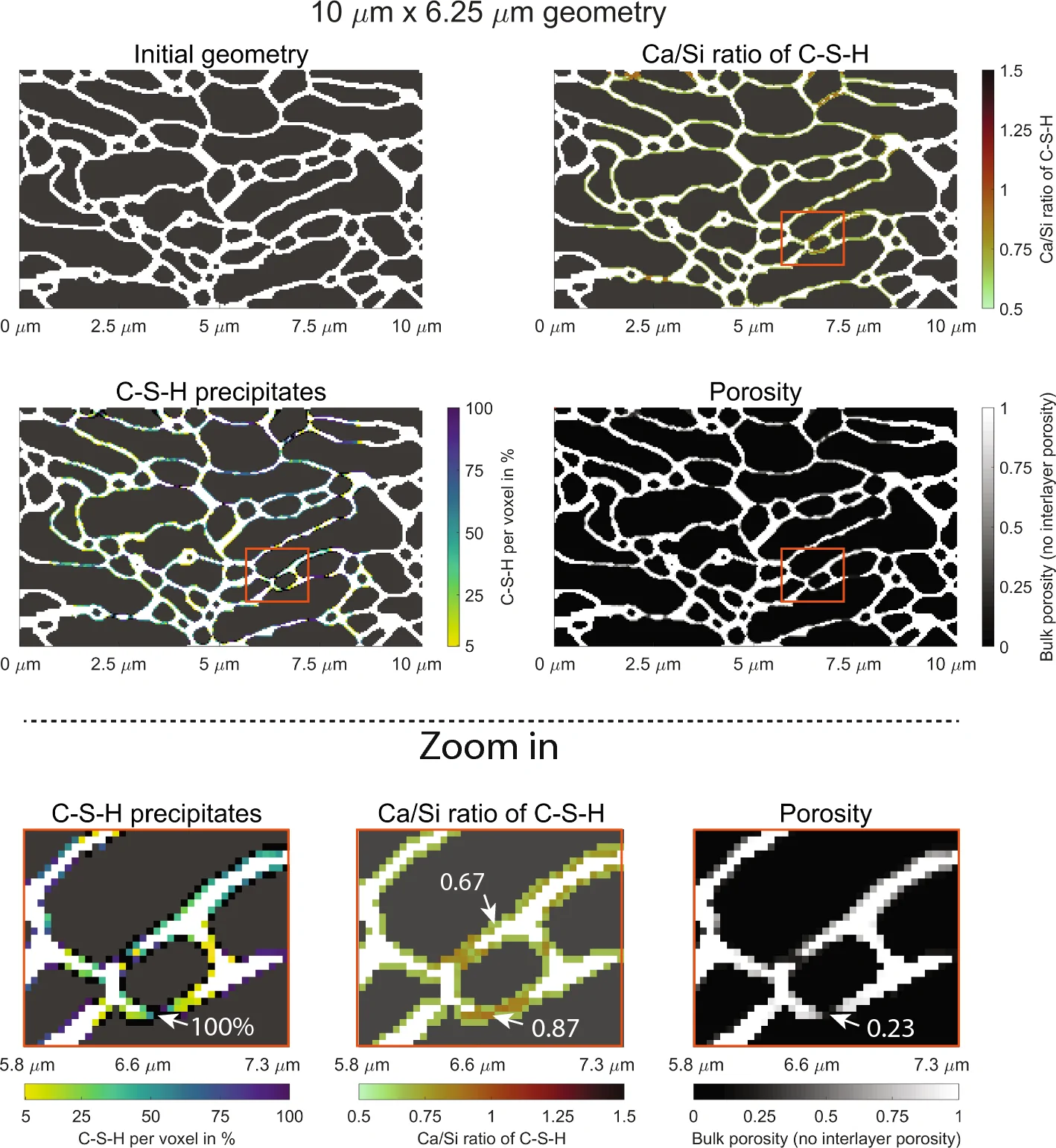
Initial geometry of the porous media for Case II with the 10 x 6.25 μm simulation domain (top, left) and the evolved ones with the colored pixels, close to clogging conditions containing information about Ca/Si ratio of C-S-H (top, right, only for voxels with more than 5% C-S-H precipitates), C-S-H precipitates (bottom, left) and porosity (bottom, right). The results stem from the simulation with a TOSS threshold of $10^{-4}$ $\frac{\%}{50\ \text {base time-steps}}$. The bottom three plots show a zoom into a descriptive section of the simulation domain. The gel porosity is not considered

Figure 11 shows the results of the simulation for Case II (while both applying the geochemical surrogate model and the adaptive time-stepping algorithm) at clogging (final state) for the most important parameters which are the local C-S-H precipitates, Ca/Si ratio of C-S-H within the pores. It is apparent that the C-S-H precipitates at the time of clogging are evenly distributed with a slight tendency toward the right side (41% in the left half, 59% on the right half) of the simulation domain. The Ca/Si ratio of the C-S-H has values between 0.67 and 0.9 (Fig. 11, top right). The voxels neighboring to solid voxels from the clay minerals that precipitated earlier typically have values of 0.67, while the voxels in the middle of the pore space that precipitated in a later stage have higher values up to 0.9. This is due to the exclusive precipitation of Tob-H at the beginning of the simulation with an increased ratio of Tob-D and Jen-H at a later stage. Jen-D only precipitated in neglectable amounts. The solidified voxels on the right side of the pore space that led to clogging have a porosity of 0.11–0.23 when the gel porosity is considered.

#### Adaptive Time-Stepping: Effect of TOSS Threshold

**Fig. 12:**
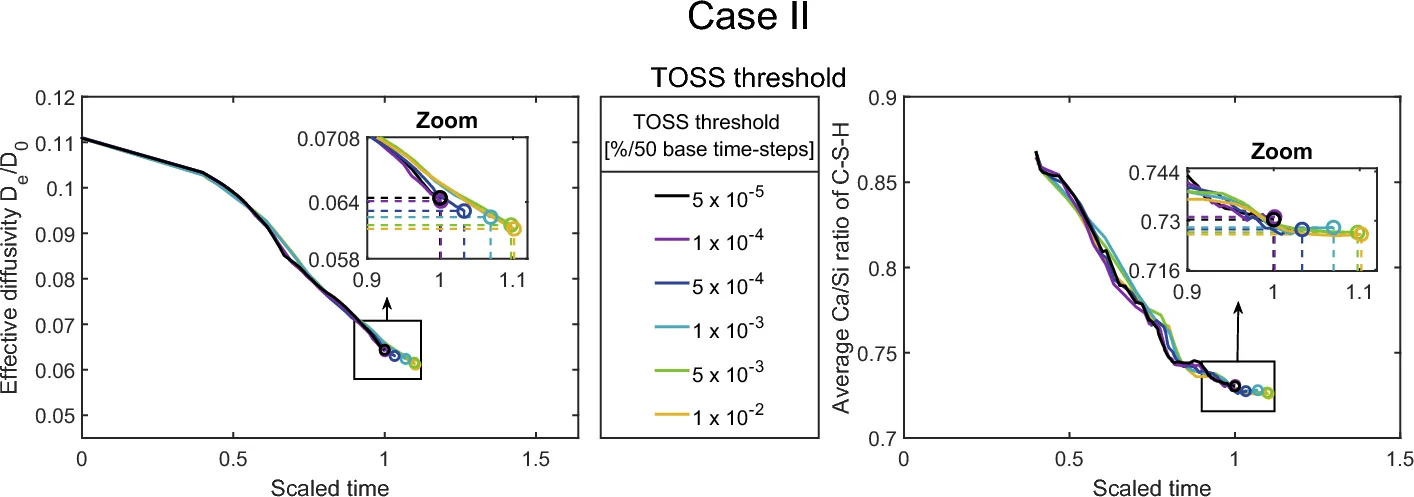
Time evolution of the effective diffusivity (left) and Ca/Si ratio of C-S-H (right) for all simulations of varying the TOSS threshold in Case II. The two lowest TOSS thresholds (black and purple curves) result in system evolutions which are in excellent agreement with each other. Higher TOSS thresholds (blue, green and yellow curves) result in slight deviations in the time evolution

The time evolution of the effective diffusivity and the average Ca/Si ratio of C-S-H for all simulations of the set of varying the TOSS thresholds for Case II and the reference simulation (red) are displayed in Fig. 12. All simulations comprise the same trend and have a close resemblance for the time evolution of the effective diffusivity and Ca/Si ratio of C-S-H. Discrepancies are mostly apparent close to the time of clogging. In general, the TOSS thresholds of $5 \cdot 10^{-5}$ and $10^{-4}$ $\frac{\%}{50\ \text {base time-steps}}$ result in simulations that have almost identical time evolutions. Higher TOSS threshold values lead to a continuous increase in time and a decrease in the effective diffusivity or Ca/Si ratio of C-S-H.

**Fig. 13:**
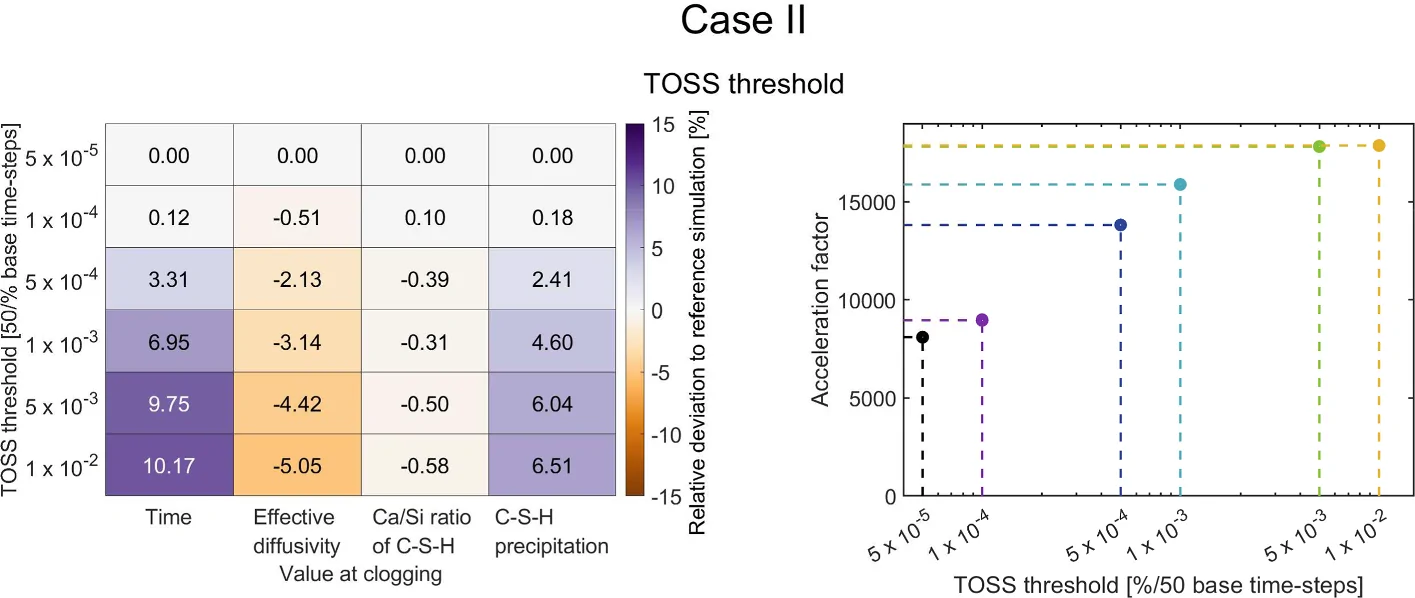
Heatmap with the relative deviations in % compared to the reference simulation for the simulation set of varying the TOSS threshold for relevant parameters at clogging (left) and plot for the acceleration factors (right) for Case II. Lower TOSS thresholds lead to higher acceleration factors, but also higher deviations from the reference simulation

The relative percentage deviations for the relevant parameters at clogging between the simulations with different TOSS thresholds and the reference simulation are displayed in a heatmap sorted by increasing threshold value (Fig. 13, left). It is important to note that the reference simulation corresponds to the simulation with the lowest tested TOSS threshold of $5 \cdot 10^{-5}$ and $10^{-4}$ $\frac{\%}{50\ \text {base time-steps}}$. The simulation with a TOSS threshold of $10^{-4}$ $\frac{\%}{50\ \text {base time-steps}}$ has low relative deviations with absolute values below 0.5% for all parameters. The absolute relative deviations increase continuously with higher TOSS thresholds. However, the degree varies between the parameters. The Ca/Si ratio of C-S-H has absolute relative deviations below 0.6% for all simulations. It goes up to 5% for the effective diffusivity, 6.5% for the C-S-H precipitation and 10.2% for the time at clogging. The simulations with the stricter TOSS thresholds of $5 \cdot 10^{-5}$ $\frac{\%}{50\ \text {base time-steps}}$ come with an impressive acceleration factor of 8’113 times and 8’978 times, respectively (Fig. 13, right). The acceleration factor achieved exceeds the mark of four orders of magnitude, and it continuously increases with higher TOSS thresholds up to 17’815 for $5 \cdot 10^{-3}$ $\frac{\%}{50\ \text {base time-steps}}$ after which it reaches a plateau.

#### Effect of Pre-activation Phase

The results of the simulations of the set of varying the pre-activation phase for Case II are presented here with more detailed plots in Supplementary information. The pre-activation phase can make up a significant portion of the total simulation duration of around 800’000 base time-steps with the implementation of the adaptive time-stepping algorithm. Analogous to Case I, the selection of the pre-activation phase allows one to balance the gains in computational time with respect to the simulation accuracy. For example, the simulation with a pre-activation phase of 200’000 base time-steps has an absolute relative deviation below 1% for all parameters at clogging. The simulation with a pre-activation phase of 100’000 base time-steps leads to absolute relative deviations of up to 4.5 and 5.2% for all parameters, except the Ca/Si ratio of C-S-H which maintains an absolute relative deviation of 0.2%. The accuracy strongly decreases for the simulation with a pre-activation phase of zero base time-steps. The time has an absolute relative deviation of 61%, the effective diffusivity and precipitation of C-S-H of 23% and the Ca/Si ratio of C-S-H of 2%.

#### Effect of the Screening Window

The results of the simulations of the set of varying the pre-activation phase for Case II are presented here with more detailed plots in Supplementary information. The simulations with screening window factors of 1, 1.11 and 1.25 have very similar time evolutions of the effective diffusivity and Ca/Si ratio of C-S-H with absolute relative deviations below 1% (compared to the simulation with a screening window factor of 1). The screening window factors of 1.43 and 1.67 show slight discrepancies with absolute relative deviations below 3%. The screening window factor of 2.5 shows well-visible discrepancies with absolute relative deviations up to 12%. The effect of the screening window on the required number of adapted time-steps and the number of solidified voxels per adapted time-step is shown in Table 6.

**Table 6:** The effect of the screening window factors in Case II on the number of adapted time-steps required to complete the RT-simulations and the average number of solidified voxels per adapted time-step

Screening window factor [-]	Number of adapted time-steps	Solidified voxels per adapted time-step
1.00	290	1.0
1.11	265	1.1
1.25	228	1.3
1.43	174	1.7
1.67	152	2.1
2.50	93	4.0

#### Porosity-Diffusivity Relation

**Fig. 14:**
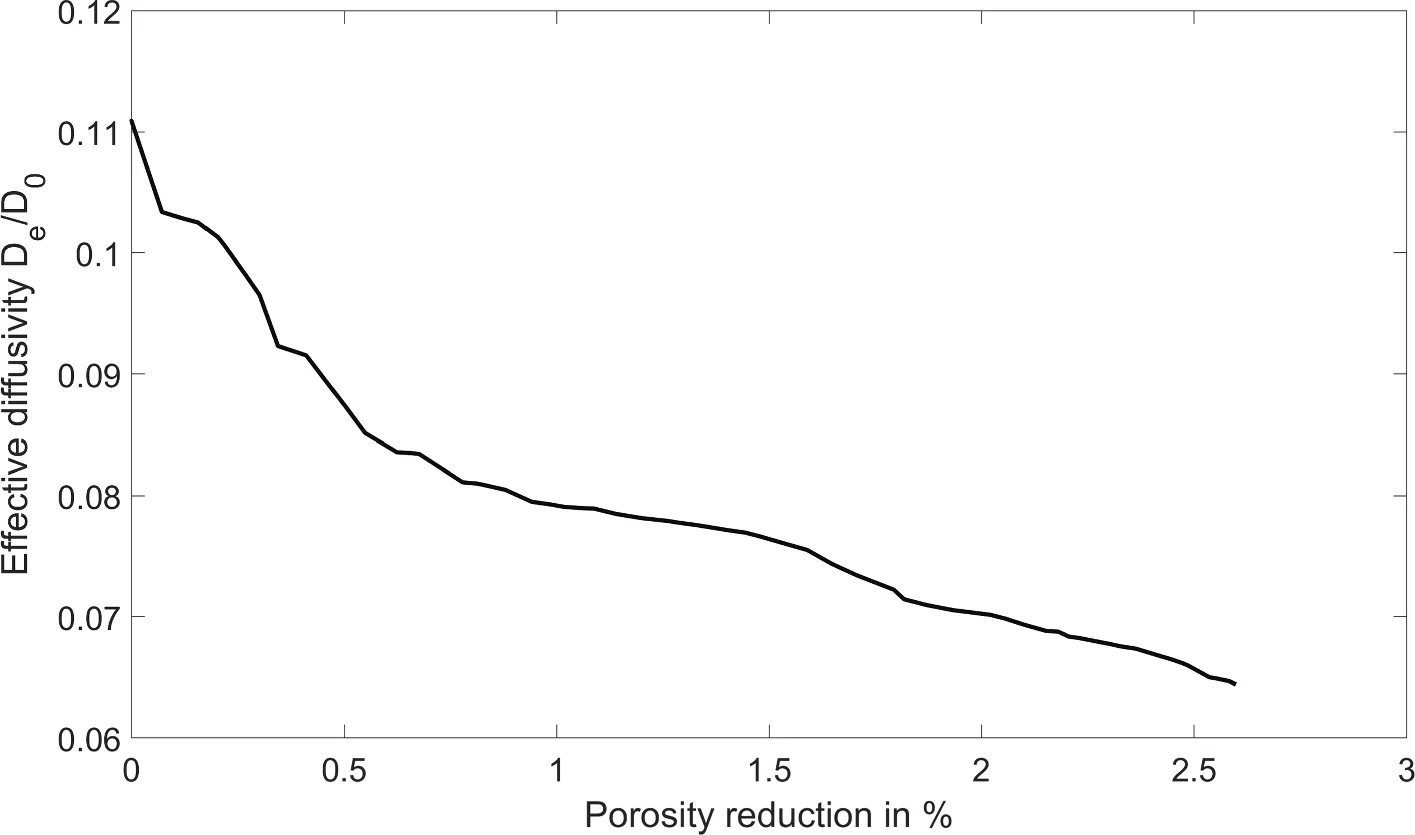
Relation between effective diffusivity and porosity reduction in % from the simulation with a TOSS threshold of $5 \cdot 10^{-5}$ $\frac{\%}{50\ \text {base time-steps}}$, pre-activation phase of 200’000 base time-steps and a screening window factor of 1.11

Figure 14 shows the correlation between effective diffusivity and reduction of porosity due to precipitation of C-S-H. The effective diffusivity changes from 0.11 (initial simulation domain) to 0.062 (simulation domain at clogging) as already visible in Fig. 10. This corresponds to a reduction of around 44%. The porosity changes from 0.274 (initial geometry) to 0.267 (geometry at clogging; the gel porosity is not considered during the RT-simulations), which corresponds to a reduction of around 2.6% of the considered simulation domain. The slope of the decreasing effective diffusivity is steeper at the beginning with a porosity reduction between zero and 0.5% (porosity between 0.274 and 0.272). The slope becomes less steep between a porosity reduction of 0.5–2.6% (porosity of 0.272 and 0.267). The porosity reduction is low when the computational domain is considered an elementary volume.

#### Grid Convergence Study

**Fig. 15:**
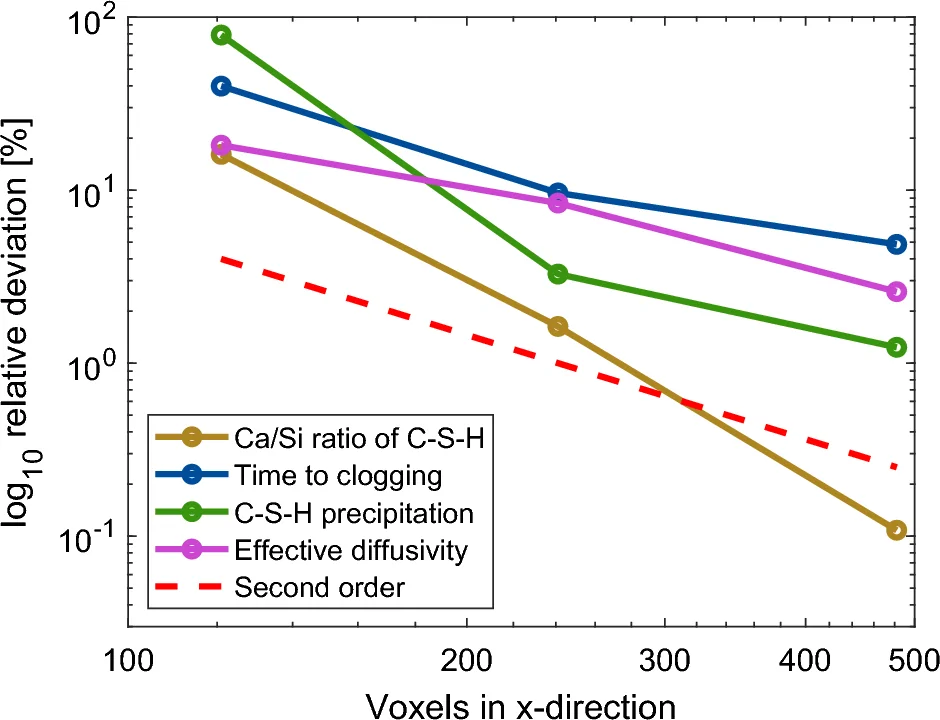
Convergence test for relevant parameters at clogging on the 10 x 6.25 μm simulation domain

A grid convergence study test was performed to identify the effect of grid resolution on the validity of the adaptive time-stepping methodology. For that, the simulation domain of 10 x 6.25 μm was resolved from coarse to fine grid as 121 x 76 (0.5x), 241 x 151 (1x) and 482 x 302 (2x) voxels. The simulations were performed on all three geometries with the initial and boundary conditions from Table 5. The TOSS threshold is set to $10^{-4}$ $\frac{\%}{50\ \text {base time-steps}}$, screening window factor to 1.11 and the pre-activation phase to 200’000 base time-steps. The relative percentage deviations for the Ca/Si ratio of C-S-H, time, C-S-H precipitation and effective diffusivity at the onset of clogging were obtained by extrapolating toward a theoretically correct value with the Richardson extrapolation technique while assuming a second-order convergence in space and time, which is typical for Lattice-Boltzmann schemes. The convergence test obtained from those results shows that the accuracies of all four parameters increase with a higher number of voxels (higher resolution). They resemble the second-order line that fits to the second order of convergence/accuracy that the LBM inherits (Fig. 15).

### Case III: RT-Simulation with Open Gel Pores for Diffusion

**Fig. 16:**
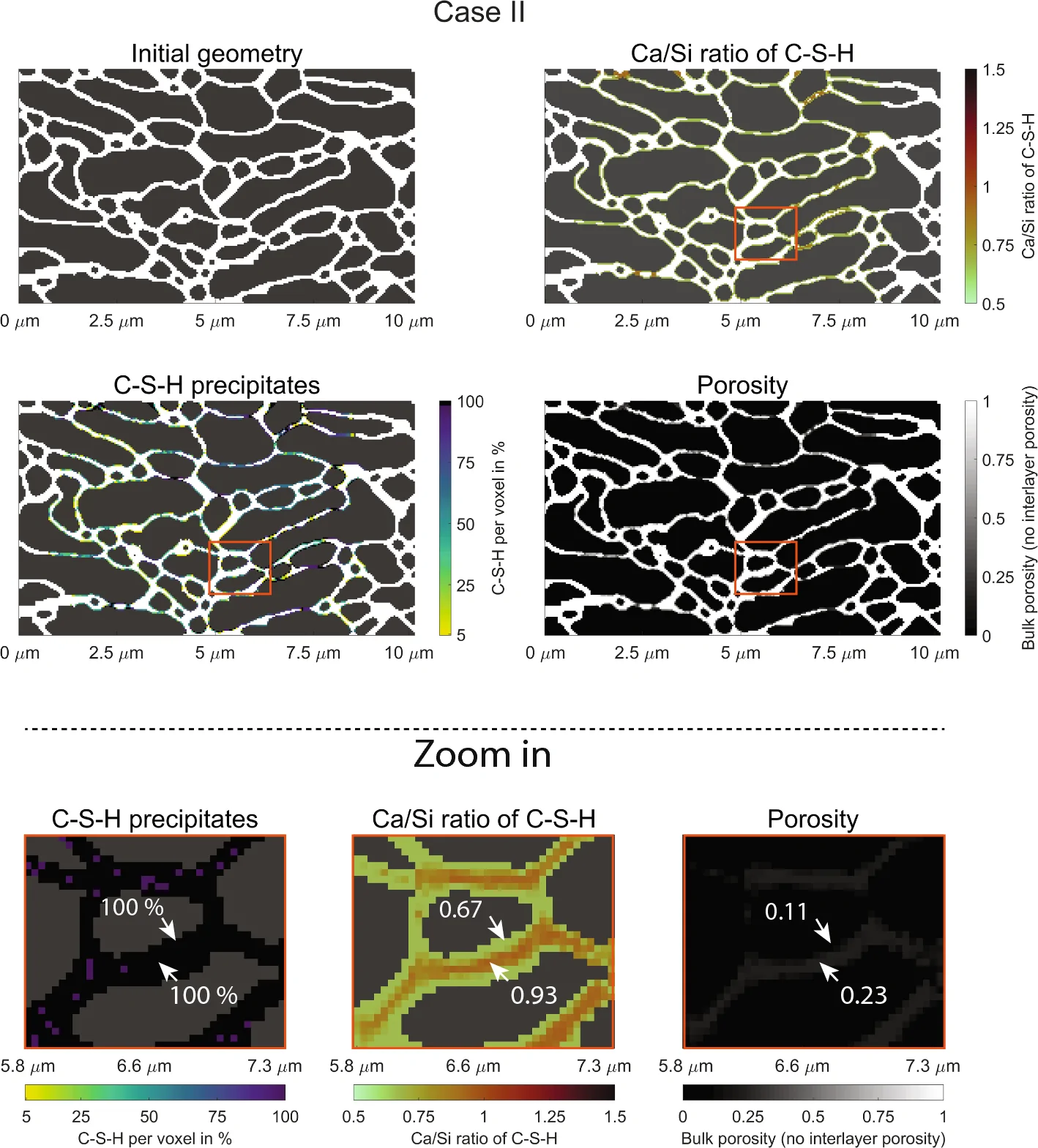
Initial geometry of the porous media in the 10 x 6.25 μm simulation domain (top, left) and the evolved ones with the colored pixels, close to clogging conditions containing information about Ca/Si ratio of C-S-H (top, right, only for voxels with more than 5% C-S-H precipitates), C-S-H precipitates (bottom, left) and porosity (bottom, right). The bottom three plots show a zoom into a descriptive section of the simulation domain. In this case, the gel porosity is taken into account

A simulation in the simulation domain of 10 x 6.25 μm (same as in Case II) was performed with the gel pores of the C-S-H precipitates open for the diffusion of $\mathrm {Ca(OH)_{2\,[aq]}}$ and $\mathrm {SiO_{2\,[aq]}}$ during the RT-simulation. The diffusion coefficient of the voxels containing C-S-H precipitates is calculated with the sub-grid model described in the subchapter Characteristic modeling parameters which is also used for the diffusion simulations in Case I and II. The adaptive time-stepping algorithm is applied with a TOSS threshold of $10^{-4}$ $\frac{\%}{50\ \text {base time-steps}}$, pre-activation phase of 200’000 base time-steps and screening window factor of 1.11. The initial simulation domain is displayed in Fig. 16 and the boundary and initial conditions are apparent in Table 3. The open gel porosity allowed the precipitation of C-S-H after clogging of the pore space as visible in Fig. 13, bottom left. Nearly all the middle part is completely filled with C-S-H with a Ca/Si ratio between 0.67 and 0.93. A zonation occurs in some pores with higher Ca/Si ratio in the middle and low ratios at the rim (Fig. 13, top right). The variation of the Ca/Si ratio of C-S-H also leads to varying local porosity along the simulation domain with values between 0.11 and 0.23 on voxels that are completely filled with C-S-H (due to the gel pores). The boundary conditions are set for fixed concentrations below the saturation of C-S-H for $\mathrm {Ca(OH)_{2\,[aq]}}$ on the right and $\mathrm {SiO_{2\,[aq]}}$ on the left side. Therefore, no precipitation occurs near the boundaries. The simulation has an acceleration factor of 12’801.

**Fig. 17:**
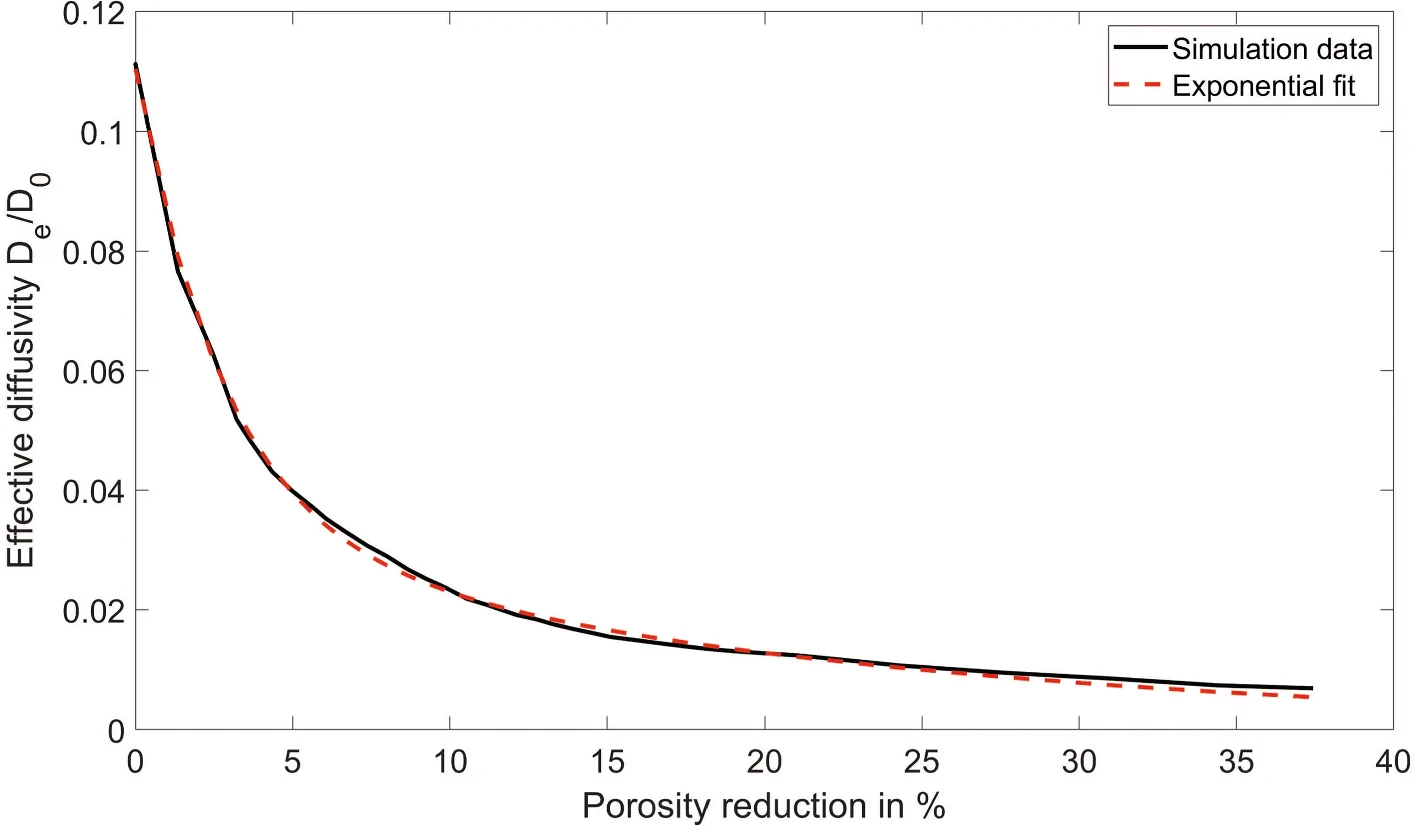
Relation between effective diffusivity and porosity reduction in % from Case III

Figure 17 shows the correlation between effective diffusivity and reduction of porosity. The effective diffusivity changes from 0.11 (initial geometry) to 0.0068 (geometry at clogging). This corresponds to a reduction of two orders of magnitude. The porosity changes from 0.274 (initial geometry) to 0.171 (geometry at clogging), which corresponds to a reduction of around 37% in the simulation domain considered.

An exponential fit to the simulation data is shown in Fig. 17. It is calculated after Eq. 21 with D_e_ being the effective diffusivity and ϵ_Reduction in %_ the reduction of porosity in %, which is calculated after Eq. 20. It is worth noting that an equation with one exponential expression or power laws was not sufficient to make an adequate fit because of the strong decrease of the effective diffusivity with further porosity reduction.

20 $$\begin{aligned} \varepsilon _{\text {Reduction in \%}}= & \left( 1 - \frac{\varepsilon _{\text {current}}}{\varepsilon _{\text {initial}}}\right) \cdot 100\% \end{aligned}$$

21 $$\begin{aligned} D_e= & 0.076 \cdot e^{-0.36 \cdot \varepsilon _{\text {Reduction in \%}}} + 0.034 \cdot e^{-0.05 \cdot \varepsilon _{\text {Reduction in \%}}} \end{aligned}$$

## Numerical Performance

### Performance of the Adaptive Time-Stepping Algorithm

#### TOSS Monitoring and Threshold Parameter

The performance and efficiency of the adaptive time-stepping algorithm are mainly determined by the definition and identification of the TOSS, which is controlled by the TOSS threshold. Such a TOSS in the scope of a RT-simulation is the temporary relaxation toward a zero rate of change of the diffusion field (described, for example, by the steady average solute flux) and of the chemical reaction rates (described by the total precipitation rate). Under such a condition, the relative percentage difference of the diffusive flux and precipitation rates compared to their values of a few previous base time-steps converges to a very small value, if not zero. The algorithm is designed to handle possible small fluctuating differences, for example, originating from the geochemistry surrogate model. It averages the values of the TOSS acceptance criteria and considers a larger number of base time-steps for the relative percentage difference. The proven functionality is demonstrated by the exceptional accuracy of the TOSS threshold values of and $5 \cdot 10^{-5}$ and $10^{-4}$ $\frac{\%}{50\ \text {base time-steps}}$ with absolute relative deviations below 2% for the parameters measured at clogging (final state of the system). An increase in the TOSS threshold provides a less strict criterion for determining the TOSS and will result in triggering the adaptive time-stepping while the diffusion and precipitation fields are still in a transient state. This leads to an increase in the deviation from the true solution. The degree of decreasing accuracy is greater for Case I than for Case II due to the following two factors. First, the chemical gradient is stronger in Case I which leads to stronger dynamics in the changing diffusion field and precipitation field. Second, the simulation domain of Case I has fewer voxels. Therefore, every erroneously solidified voxel can lead to larger deviations in the diffusion and precipitation field from the true solution. Both factors cause Case I (or generally reactive transport systems with a smaller simulation domain and higher chemical gradients) to respond more sensitively to a premature triggering of TOSS. However, not all parameters at clogging have the same sensitivity to the TOSS threshold. Generally, the absolute relative deviations from the reference simulation in the final state after increasing the TOSS threshold are highest for the time (up to 17%), moderate for the effective diffusivity and C-S-H precipitation (up to 7%) and lowest for the average Ca/Si ratio of C-S-H (up to 4%). Although the accuracy of the adaptive time-stepping algorithm decreases with higher TOSS threshold values, the acceleration factor improves. In both Case I and II, the TOSS threshold of the order $10^{-4}$ $\frac{\%}{50\ \text {base time-steps}}$ has the right balance between the achieved accuracy (absolute relative deviation below 2.3%) and efficiency (acceleration factor of 90 in Case I and 8’978 in Case II). This TOSS threshold value should be applicable for RT-simulations with similar concentration gradients and simulation domain size.

#### Initial Transient State Identification

A premature determination of the first TOSS (after the initial transient state) seems to have a crucial impact on the total outcome, because potential errors can propagate throughout the simulation time. It is apparent from the results of Case I and II that the simulations without a pre-activation phase have large inaccuracies. For example, the relative deviation of the clogging time is 8% for Case I and 61% for Case II. This demonstrates the necessity of a controlling algorithm parameter at the beginning of the simulation to avoid essentially the activation of an adapted step during the initial transient state. The simple delay of starting the monitoring by the adaptive time-stepping algorithm with a pre-activation phase is a simple solution. The pre-activation phase specifies the number of base time-steps at the beginning of the RT-simulation during the initial transient state without any actions of the adaptive time-stepping algorithm. The setting of a lower TOSS threshold at the beginning of the simulation would be another option. However, lower TOSS thresholds can be more strongly influenced, for example, by any possible small fluctuations rising from the geochemical surrogate model. This would have an effect on the accurate monitoring of the TOSS. A pre-activation phase of 200’000 base time-steps reduced the absolute relative deviations from the reference simulation to below 2% for all tested parameters at clogging for both Case I and Case II. Therefore, this value for the pre-activation phase seems to be optimal for similar reactive transport systems, which is typically the phase of strong initial transient states due to simulation initialization.

Two factors will mostly influence the extent of the initial transient state and therefore also the necessary number of base time-steps of the pre-activation phase for obtaining a good accuracy. First, it is plausible that the number of base time-steps should be increased for larger simulation domains because the diffusion time to balance the chemical gradient grows exponentially with increasing diffusion length. This can also explain the larger deviations in Case II with lower pre-activation phases because it has a larger simulation domain than Case I. Second, the initial transient state is strongly influenced by the initial conditions. It is reminded here that the bulk and the boundary conditions are set off-equilibrium at the beginning of the simulations, which lead to strong initial transient states. The pre-activation phase can be reduced or even disregarded by initializing the bulk concentrations with preexisting balanced chemical gradients, which would avoid the initial transient state.

#### Screening Window

The screening window factor controls the extent of the potential increase of the adapted time step to solidify more than one voxel per application of an adapted time-step. Such a consideration is necessary when simulation domains of the order of 1 billion voxels are considered for more resolved, complex, and/or 3-dimensional geometries. Applying the adaptive time-stepping scheme for each and every solidifying voxel would be clearly inefficient and might not be the optimal strategy due to the large number of voxels and the lower significance of each separate voxel for the total system evolution. It was observed that the implementation of the screening window decreased the total number of adapted time-steps. In Case I, the screening window factor of 1.67 reduced the necessary number of adapted time-steps to 35 instead of 96, while solidifying on average 3.2 voxels per adapted time-step. In Case II, the screening window factor of 1.25 reduced the necessary number of adapted time-steps to 228 instead of 290, while solidifying in average 1.6 voxels per adapted time-step. There was no significant influence on the accuracy of this scheme for the tested screening window factors. More tests will be needed for 3-dimensional geometries. The handling of missing heterogeneous precipitation due to solidifying several voxels in one adapted time-step has a larger influence on the performance of 3-D simulations because it potentially affects 26 voxels instead of 8 (in 2-D).

### Final Considerations of the Adaptive Time-Stepping Algorithm

As shown in the subchapter Grid convergence study of the results for Case II, the proposed scheme, including the adaptive time-stepping algorithm, maintains the second-order spatial accuracy of the Lattice Boltzmann scheme. Furthermore, the strictness of the acceptance criteria has an influence on the accuracy and efficiency of the RT-simulations. By considering their physical meaning and the observations so far, it is possible to discuss the guidelines for the generalization of the scheme in setups that are characterized by the existence of TOSS. Generally, the adaptive time-stepping scheme is not limited to pore-scale RTMs for the cement–claystone interface. It can be applied to any kind of reactive transport system that is characterized by phenomena evolving at multiple time scales if there is a clear separation of scales and where TOSS exists. The advection–diffusion-reaction microfluidic setup of (Poonoosamy et al. 2019) is such an example. The adaptive time-stepping scheme is especially efficient for reactive transport systems with low reaction rates. This is demonstrated by the varying acceleration factors between Case I and II. Case II has around 100 times lower precipitation rate per voxel and base time-step than Case I, leading to almost 100 times higher acceleration factors (around 90 in Case I and 9’000 in Case II) by the adaptive time-stepping scheme.

### Geochemical Surrogate Model

The geochemical surrogate model used in Case I accelerates the geochemical calculations by 41 times, and the model used in Case II accelerates it by 54 times compared to the calculations with the geochemical solver. The difference most probably occurs from the different neural network architectures. (More neurons and hidden layers lead to higher computational cost.) The one-time effort for training and the trial-and-error approach to find the optimal neural network architectures must also be considered. However, the training cost is relatively small when the gains in overall simulation time are considered. Accelerated RT-simulations now take a few hours compared to weeks or months when the geochemical surrogate model is considered. Once trained, the same geochemistry surrogate model can be used for several types of investigations, for example, in the sensitivity analysis of the influencing parameters or in a test of different initial and boundary conditions in the same RTM case.

Considering the additional advantage of employing surrogate models for the chemistry, compared to a traditional simulation coupled directly to the geochemical solver, it leads to an overall combined acceleration factor of 4’950 for Case I and 484’812 for Case II while considering the computational acceleration of the adaptive time-stepping algorithm and the geochemical surrogate model. It is even higher for Case III with an overall acceleration factor of around 700’000.

## Application of the Modeling Results to the Cement–Claystone Interface Reactivity

Mineral transformations and the temporal evolution of transport properties at the cement–claystone interface has been monitored for several years in a dedicated laboratory experimental setup at the mm-scale (Luraschi et al. 2020; Shafizadeh et al. 2020). The low porosity zone of mostly C-S-H precipitates formed in the claystone side with an extent of 1–2 mm across the interface. The effective diffusivity of the low porosity zone decreased by almost two orders of magnitude for uncharged molecules (measured with HTO) and three orders of magnitude for anions (measured with ^36^Cl^-^). The HTO flux has never reached zero, indicating the everlasting open gel porosity and its connectivity similar to Case III. The importance of the cement–claystone interface accessibility for mass transport is elaborated in the chapter Introduction. The relevance of C-S-H precipitates, which ensure the everlasting mass transport even though the macropores appear clogged is further corroborated by the RT-simulations in this paper. In Case I and II, in which the gel pores were considered as inaccessible for the diffusion of aqueous solutes, the macropores are filled with precipitates and clogged relatively fast, with a short extent of the low porosity zone of less than 1 μm and a porosity reduction of around 2.5% for the whole simulation domain. In these cases, the two sides of the domain are disconnected. The remaining gel porosity due to C-S-H precipitation is considered in Case III, where the gel pores were accessible for diffusion of all aqueous solutes. This resulted in the low porosity zone extending over the simulation domain of 10 μm with a resulting porosity reduction of 35% for the assigned boundary conditions and simulation domain size. The same cementation factor of 2 for calculating the effective diffusivity of the subgrid C-S-H gel porosity as computed in the mentioned experiments of Luraschi et al. (2020) also results in a decrease in the effective diffusivity of two orders of magnitude after the formation of the low porosity zone in the RT-simulation of Case III, which is in accordance with the experimental results.

The consideration of the precipitation of C-S-H as a solid solution enables a further complementary understanding of the pore-scale processes of the laboratory and long-term experimental investigations. It allows the calculation and tracing of the Ca/Si ratio within the precipitated C-S-H, resulting in variable nanostructure characteristics like the local gel porosity. This finally leads to the observation of the characteristics of the spatial and temporal heterogeneity of the Ca/Si ratio and gel porosity of the C-S-H precipitates during the reactive transport. For example, the Ca/Si ratio of C-S-H ranges from 0.7 to 1.4 in Case I and from 0.66 to 0.93 in Case II and III, indicating a strong heterogeneity and dependence on in situ conditions. The C-S-H precipitates in Case III also show a zonation of different Ca/Si ratio in the precipitation occurring in a single pore throat, which is a very interesting physical phenomenon.

So far, this pore-scale RTM considers a spatial domain of several micrometers, while the low porosity zone is expected to span a few millimeters. The porosity–diffusivity relation of 21 derived from the RT-simulation in Case III can be used for upscaling to macroscopic models to obtain primary estimations about the characteristics of the low porosity zone. In upcoming investigations, the unprecedented computational acceleration will also enable the performance of numerous pore-scale RT-simulations with different boundary conditions and simulation domains to obtain more sophisticated correlations for upscaling. The presented acceleration methods can also be combined with GPU parallelization which allows RT-simulations in simulation domains discretized by several billion voxels, allowing to consider a larger simulation domain up to the mm-scale.

The pore-scale RTM presented here can be used to simulate different scenarios of the diffusion of cementitious pore water into the claystone macropores. The simulation domain and degradation stage can be adapted, or different cement recipes or clay minerals can be investigated. The underground repositories for radioactive waste disposal require a planning and optimization phase, which is crucial for its feasibility and safety aspects. Therefore, it is beneficial to have a supportive tool which can consider different scenarios to complement laboratory and long-term experimental investigations. An extension to the model would be the inclusion of electrostatic considerations and the implementation of the Nernst–Planck equations to ensure charge balance due to the interactions of the ions with the charged surface of C-S-H (Yang et al. 2019) and the clay minerals (Jenni et al. 2017). This mechanism is expected to have an influence since the C-S-H gel porosity surfaces are typically charged, and the pores have a dimension which can induce effects such as the limited anion accessibility. Furthermore, different chemical reactions are expected to occur in addition to the C-S-H precipitation, which depends on the mineralogical composition of the claystone at hand (Kosakowski and Berner 2013; Wilson et al. 2021). This is important because even thin films of precipitates from other solid phases on the C-S-H surface can significantly affect the evolution of the microstructure and solute transport pathways by blocking the access of the aqueous solutes or gas molecules to the gel pores. An accurate model of such potential phenomena requires RTM at the pore scale for considering the distribution of the individual solid phases within the pores, such as the model presented here.

It is also typical to use the equilibrium approach instead of kinetics for the C-S-H precipitation. The equilibrium approach is clearly defined, computationally faster and easier to implement than the kinetics approach, especially when considering that sometimes the reaction pathways and kinetic rates of intermediate reactions are not known. However, it might come with some shortcomings in cases where the evolution of the system is kinetically controlled rather than mass transport controlled. The consideration of kinetics could be a further extension to the model presented here. RTMs using the kinetic approach (Trapote-Barreira et al. 2016; Chaparro et al. 2017; Löher et al. 2025) or kinetic experiments (Baur et al. 2004; Trapote-Barreira et al. 2014) document reaction rates of C-S-H precipitation in a range of 10^− 13^ to 10^− 7^ mol s^− 1^ m^− 2^. An exact experiment matching the evolving in situ conditions, which measures the involved reaction kinetic rates, does not exist to the best of our knowledge. The reactive transport model of this paper using the equilibrium approach is characterized by average precipitation rates per voxel in the reactive zone of $2.6 \cdot 10^{-5}$ to $2.2 \cdot 10^{-6}$ mol s^− 1^ m^− 2^ for Case I and $1.1 \cdot 10^{-7}$ to $3.6 \cdot 10^{-9}$ mol s^− 1^ m^− 2^ for Case II and III. This calculation assumes a flat surface of the C-S-H precipitates for the minimal value and the surface of C-S-H of a voxel filled with C-S-H for the maximum value (Suda et al. 2015). More details about the calculation of the reaction rates are shown in Supplementary information. The reaction rates from the RT-simulations have values inside the range from literature for Case II and III, and they are close for Case I. Therefore, the equilibrium approach is a valid assumption for the performed RT-simulations without compromising the generality of the methodology.

## Conclusion

The central point of this work is the simulation of precipitation processes at the pore scale, relevant to the cement–claystone interface. Such simulations typically are accompanied by an extreme computational cost. An adaptive time-stepping scheme was combined with geochemical surrogate models to accelerate the simulations with minimal loss of accuracy. The developed adaptive time-stepping algorithm monitors the solute flux and reaction rates to detect TOSS. The time-consuming simulation of chemical reactions and diffusion is skipped at all detected TOSS by applying an increased time-step on the solid growth to solidify at least one voxel. The accuracy and efficiency of the algorithm are controlled by three parameters, which must be set prior to initialization of the RT-simulation. The pre-activation phase delays the activation of the adaptive time-stepping algorithm to prevent propagating errors arising from applying premature adapted time-steps during the initial transient state. The TOSS threshold sets the criterion for determining TOSS and has the largest influence on the accuracy and acceleration factor. The solidification of several voxels per time-step is implemented with the screening window, and the screening window factor sets the possible extent of the adapted time-step to solidify more than one voxel. Suggestions for parameter values are given which are valid for RTMs with similar chemical and physical conditions. It demonstrates the increased efficiency of RTMs at the pore scale with very little decrease in accuracy while properly setting the mentioned algorithm parameters. This leads to acceleration factors of two to four orders of magnitude for the two tested cases. The computational acceleration factor is generally higher for systems with lower reaction rates per base time-step.

The adaptive time-stepping algorithm was successfully applied to reactive transport systems within the pore scale that simulate the C-S-H precipitation in a claystone due to interaction with cement. This RTM acts as a foundation that can be extended to consider a chemical system that further approximates the reality of the cement–claystone interaction. It was demonstrated that it can qualitatively describe the relevant phenomena identified by experimental investigations. The two applied geochemical surrogate models accelerate the overall RT-simulations by factors of almost two orders of magnitude. Combined with the adaptive time-stepping algorithm, it leads to acceleration factors of four to six orders of magnitude. It is even higher for simulations considering the gel porosity with an overall acceleration factor of approximately 700’000 times. The presented cases demonstrate the high efficiency of this high-performance RTM while applying the adaptive time-stepping scheme and using geochemical surrogate models. This unprecedented efficiency advances the possibilities of modeling reactive transport processes at the pore scale of cement–claystone interactions and to conduct sensitivity analysis, inverse modeling and optimization investigations. The algorithmic developments are general and can also be applied to other material interfaces and systems in future investigations.

## Supplementary Information

Below is the link to the electronic supplementary material.

Supplementary file 1 (pdf 1714 KB)
