# Supplementary material for: Adaptive Time-Stepping and Machine Learning Approach for Pore-Scale Reactive Transport Simulations
Source: Transp Porous Media. 2026 Feb 12;153(3):37. doi: 10.1007/s11242-026-02287-4 (PMC13661654; doi:10.1007/s11242-026-02287-4)
Supplement: Supplementary file 1 [file 11242_2026_2287_MOESM1_ESM.pdf]

Supplementary Materials for 'Adaptive Time-Stepping and Machine Learning Approach for Pore Scale Reactive Transport Simulations'

Micha P. Baur[1,2*], Sergey V. Churakov[1,2] and Nikolaos Prasianakis[1*]

[1*]Institute of Geological Sciences, University of Bern, Baltzerstrasse 1+3, Bern, 3000, Bern, Switzerland.
[2]Laboratory for Waste Management, Paul Scherrer Institute, Forschungsstrasse 111, Villigen, 5232, Aargau, Switzerland.

*Corresponding author(s). E-mail(s): micha.baur@unibe.ch; nikolaos.prasianakis@psi.ch;

1 Adaptive time-stepping algorithm parameters for all RT-simulations

Table 1 Set of varying TOSS threshold

Run	TOSS threshold $[\frac{\%}{50 \text{ base time-steps}}]$	Pre-activation phase [base time-steps]	Screening window factor [-]
1.1	$5.0 \cdot 10^{-5}$	200'000	1.11
1.2	$1.0 \cdot 10^{-4}$	200'000	1.11
1.3	$5.0 \cdot 10^{-4}$	200'000	1.11
1.4	$1.0 \cdot 10^{-3}$	200'000	1.11
1.5	$5.0 \cdot 10^{-3}$	200'000	1.11
1.6	$1.0 \cdot 10^{-2}$	200'000	1.11

Table 2 Set of varying the pre-activation phase

Run	TOSS threshold $[\frac{\%}{50 \text{ base time-steps}}]$	Pre-activation phase [base time-steps]	Screening window factor [-]
2.1	$1.0 \cdot 10^{-4}$	300'000	1.11
2.2	$1.0 \cdot 10^{-4}$	200'000	1.11
2.3	$1.0 \cdot 10^{-4}$	100'000	1.11
2.4	$1.0 \cdot 10^{-4}$	0	1.11

Table 3 Set of varying the screening window factor

Run	TOSS threshold $[\frac{\%}{50 \text{ base time-steps}}]$	Pre-activation phase [base time-steps]	Screening window factor [-]
3.1	$1.0 \cdot 10^{-4}$	200'000	1.00
3.2	$1.0 \cdot 10^{-4}$	200'000	1.11
3.3	$1.0 \cdot 10^{-4}$	200'000	1.25
3.4	$1.0 \cdot 10^{-4}$	200'000	1.43
3.5	$1.0 \cdot 10^{-4}$	200'000	1.67
3.6	$1.0 \cdot 10^{-4}$	200'000	2.50

2 Case dependent calculations of the acceleration factor and relative devation

The effective diffusivity and average Ca/Si ratio of C-S-H are the principal results of interest which are also selected to compare simulations within each set of varying parameters in Case I and II with the respective time-evolution. Furthermore, the results at clogging (final state) are used to compare the RT-simulations by calculating the absolute relative deviation (Eq. 1) of the value from a RT-simulation at clogging ($\text{Value}_{[i]}$) to the value at clogging from the reference simulation ($\text{Value}_{[0]}$). In Case I, the reference simulation corresponds to the RT-simulation without enabling the adaptive time-stepping algorithm. In Case II, the reference simulation corresponds to the RT-simulation with the most relaxed parameter within the set of varying values of the corresponding parameter because a RT-simulation without the adaptive time-stepping would take six years on a single core (i9/14900KF CPU).

$$\text{Relative deviation (\%)} = \left| \frac{\text{Value}_{[i]} - \text{Value}_{[0]}}{\text{Value}_{[0]}} \right| \cdot 100\% \quad (1)$$

In Case I, the acceleration factors of the individual RT-simulations are calculated by dividing the required number of base time-steps to complete the respective RT-simulation by the number of base time-steps required to complete the reference RT-simulation (without the adaptive time-stepping algorithm). In Case II, the acceleration factors of the individual simulations are calculated by dividing the required number of base time-steps to complete the respective simulation by the virtual base time-step

count of the reference simulation. The virtual base time-step count corresponds to the virtual number of base time-steps skipped by all acceleration time-steps added to the actual number of simulated base time-steps.

3 Geochemical surrogate model efficiency

The computational acceleration factor of the surrogate model within the RTM is calculated according to Eq. 2.

$$\text{Acceleration factor}_{Surrogatemodel} = \frac{\text{GEMS runtime}_{10^4 runs}}{\text{Surrogate model runtime}_{10^4 runs}} \tag{2}$$

Comparisons were performed on a single core of an Intel Core i9/14900KF CPU. The geochemical surrogate model that is used in Case I required 0.0082 s for 10^4 runs, while GEMS required 0.3362 s for 10^4 runs. Therefore, this neural network leads to an acceleration of 41 times (excluding the acceleration factor of the adaptive time-stepping algorithm).

The geochemical surrogate model that is used in Case II and III required 0.0063 s for 10^4 runs, while GEMS required 0.3362 s for 10^4 runs. Therefore, this neural network leads to an acceleration of 54 times (excluding the acceleration factor of the adaptive time-stepping algorithm).

For both surrogate models, the training of the neural networks required a one-time expense of 1 h without considering the network hyperparameter optimisation effort, which depends on the approach and expert judgement and could result in several training trials.

4 Results of the simulations with varying adaptive time-stepping parameters

This section provides additional plots for Case I and II for the pre-activation phase (Fig. 1-2 and 5-6) and screening window factor (Fig. 3-4 and 7-8). Furthermore, the results from a supplementary case are shown (Table 4-5 and Fig. 9-15).

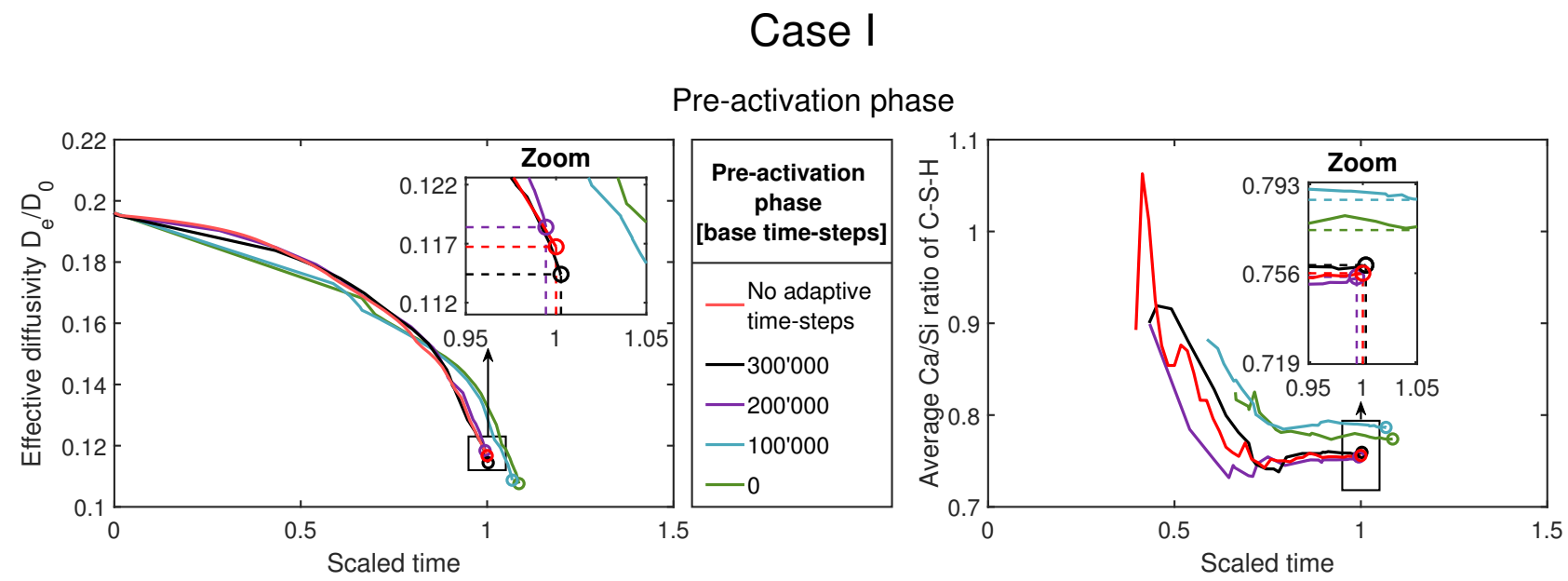

Fig. 1 Time evolution of the effective diffusivity (left) and Ca/Si ratio of C-S-H (right) for all simulations of varying the pre-activation phase for Case I.

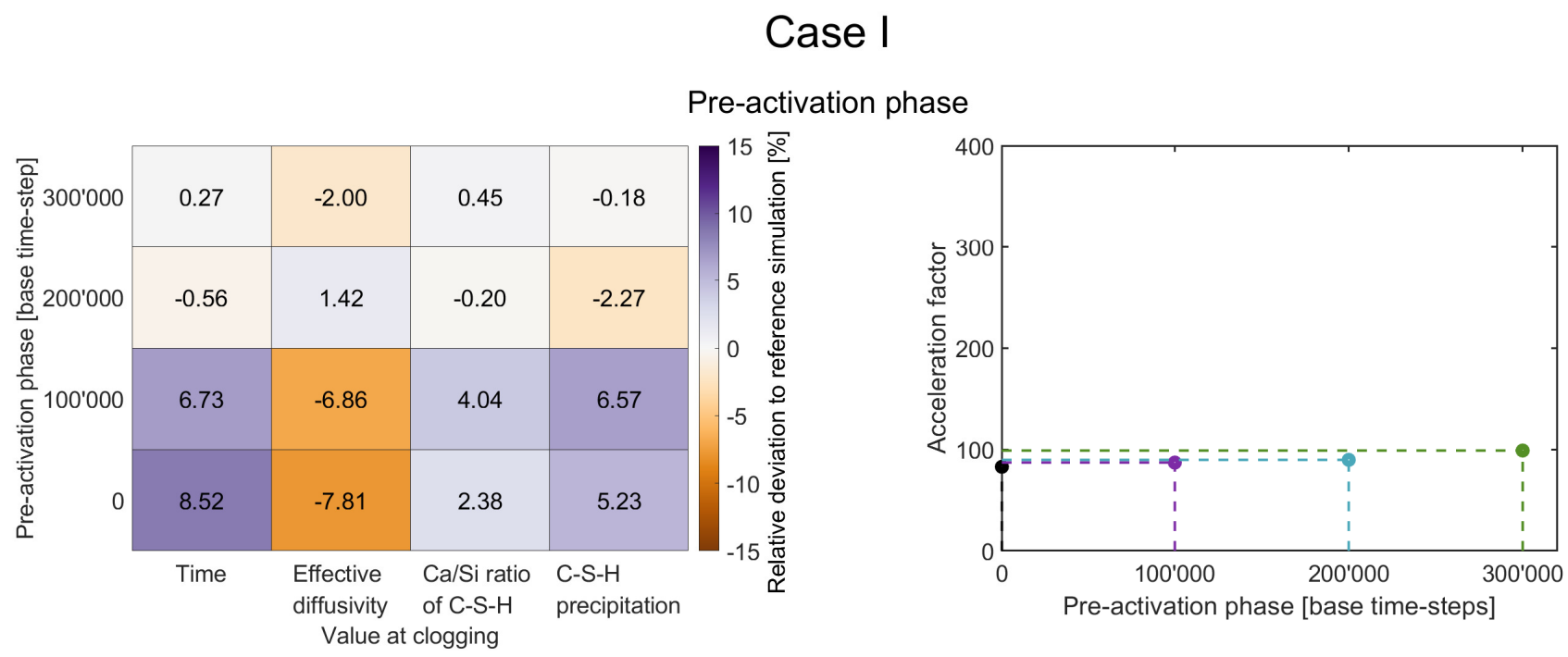

Fig. 2 Heatmap with the relative deviations compared to the simulation without the adaptive time-stepping algorithm for the simulation set of varying the pre-activation phase for relevant parameters at clogging (left) for Case I. Plot for the acceleration factors (right).

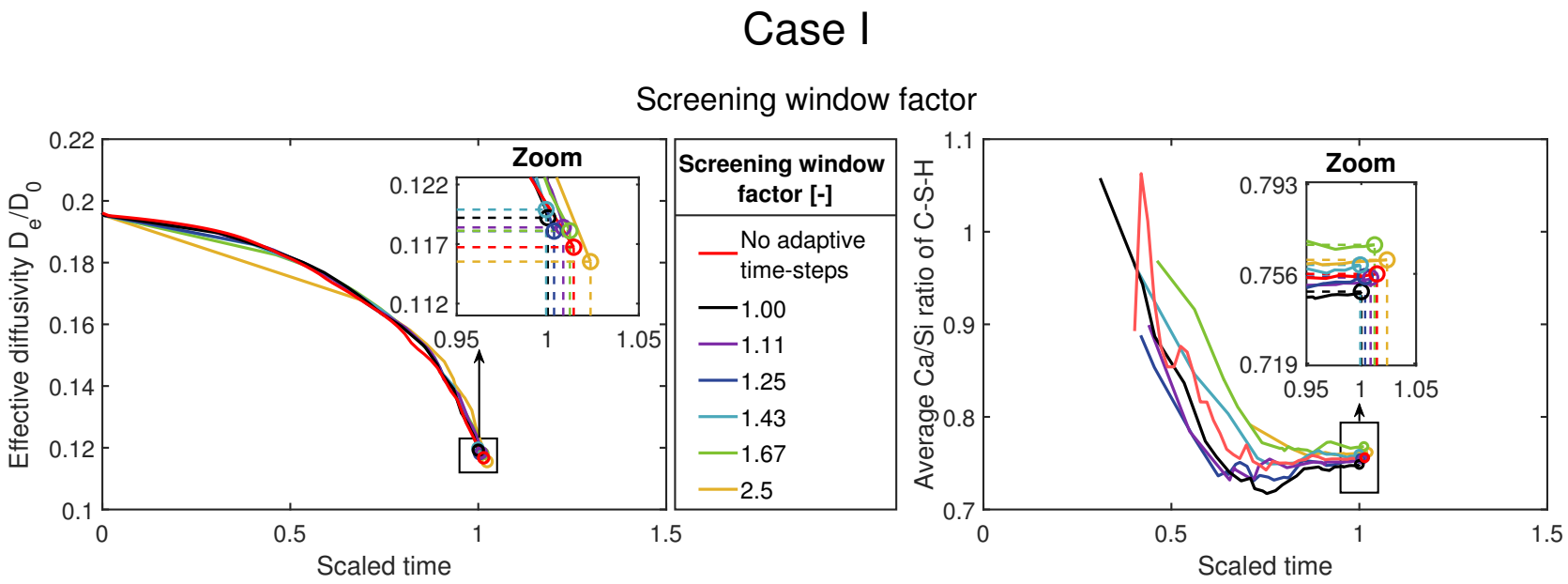

Fig. 3 Time evolution of the effective diffusivity (left) and Ca/Si ratio of C-S-H (right) for all simulations of varying the screening window factor for Case I.

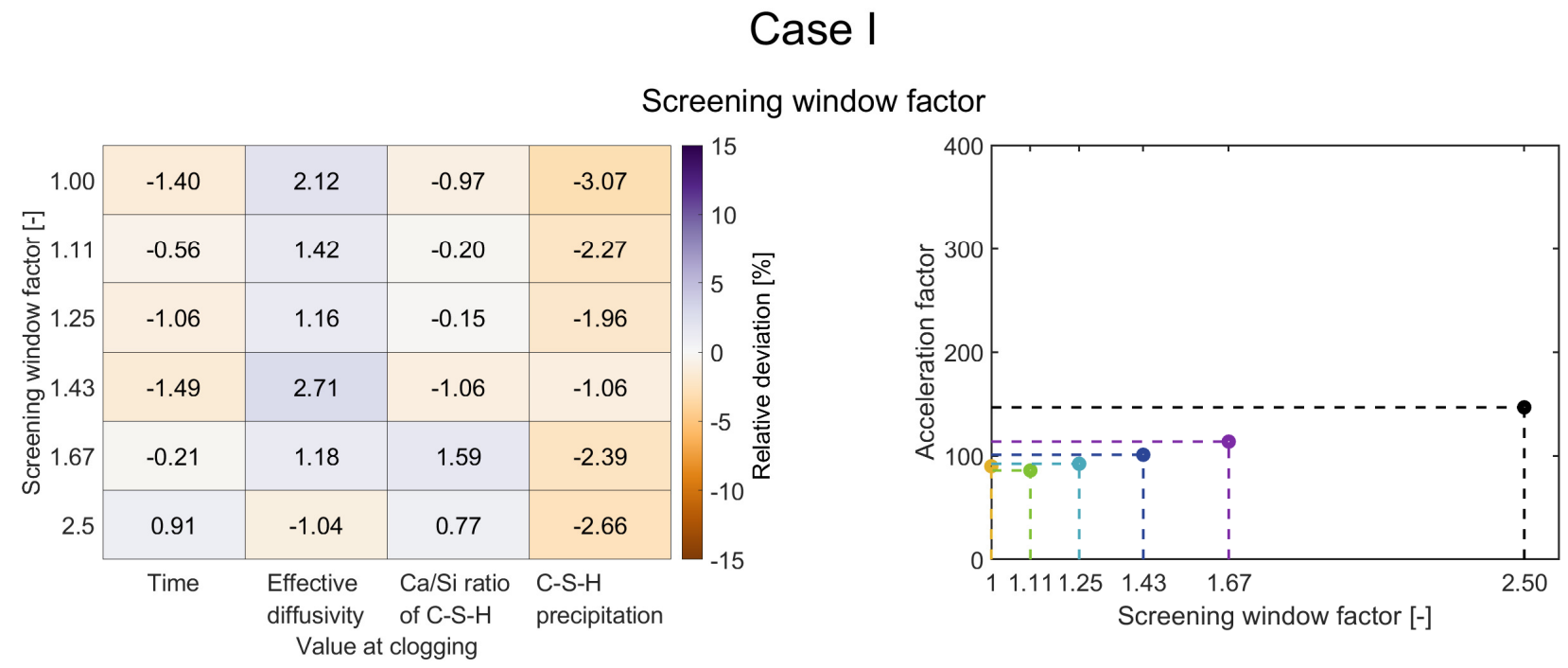

Fig. 4 Heatmap with the relative deviations compared to the simulation without the adaptive time-stepping algorithm for the simulation set of varying the screening window factor for relevant parameters at clogging (left) for Case I. Plot for the acceleration factors (right).

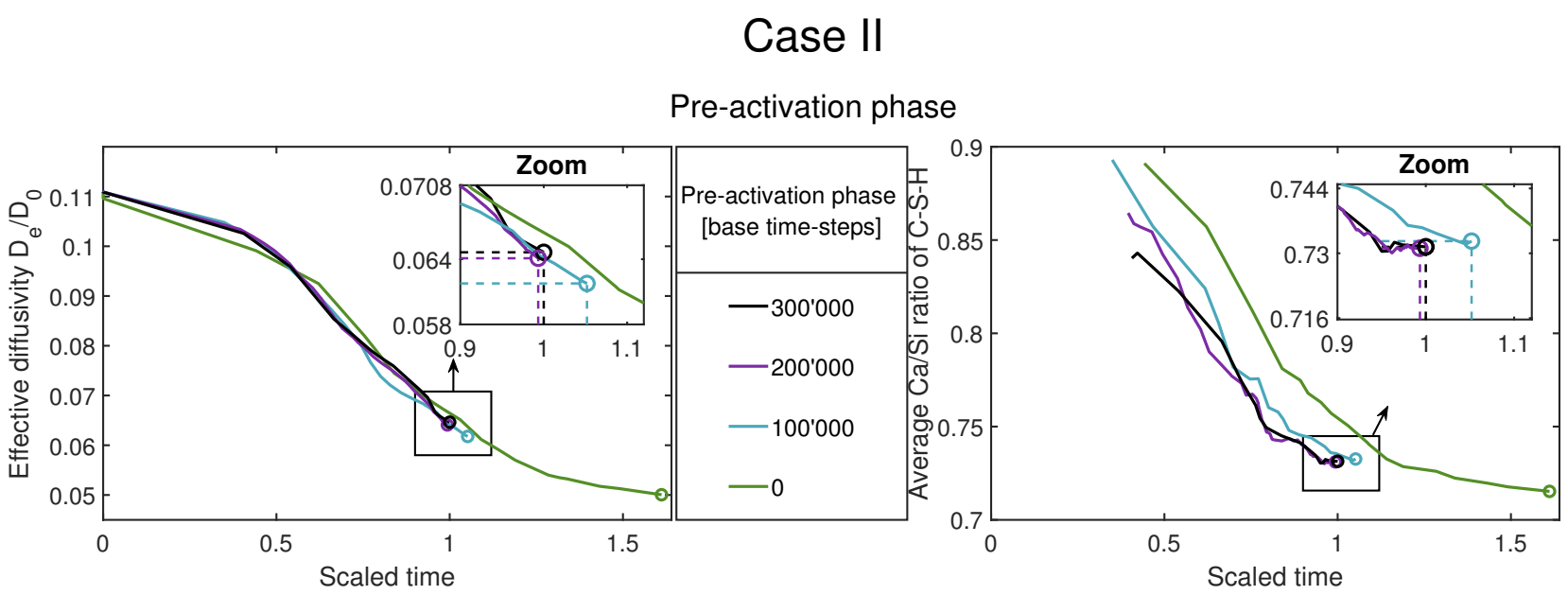

Fig. 5 Time evolution of the effective diffusivity (left) and Ca/Si ratio of C-S-H (right) for all simulations of varying the pre-activation phase for Case II.

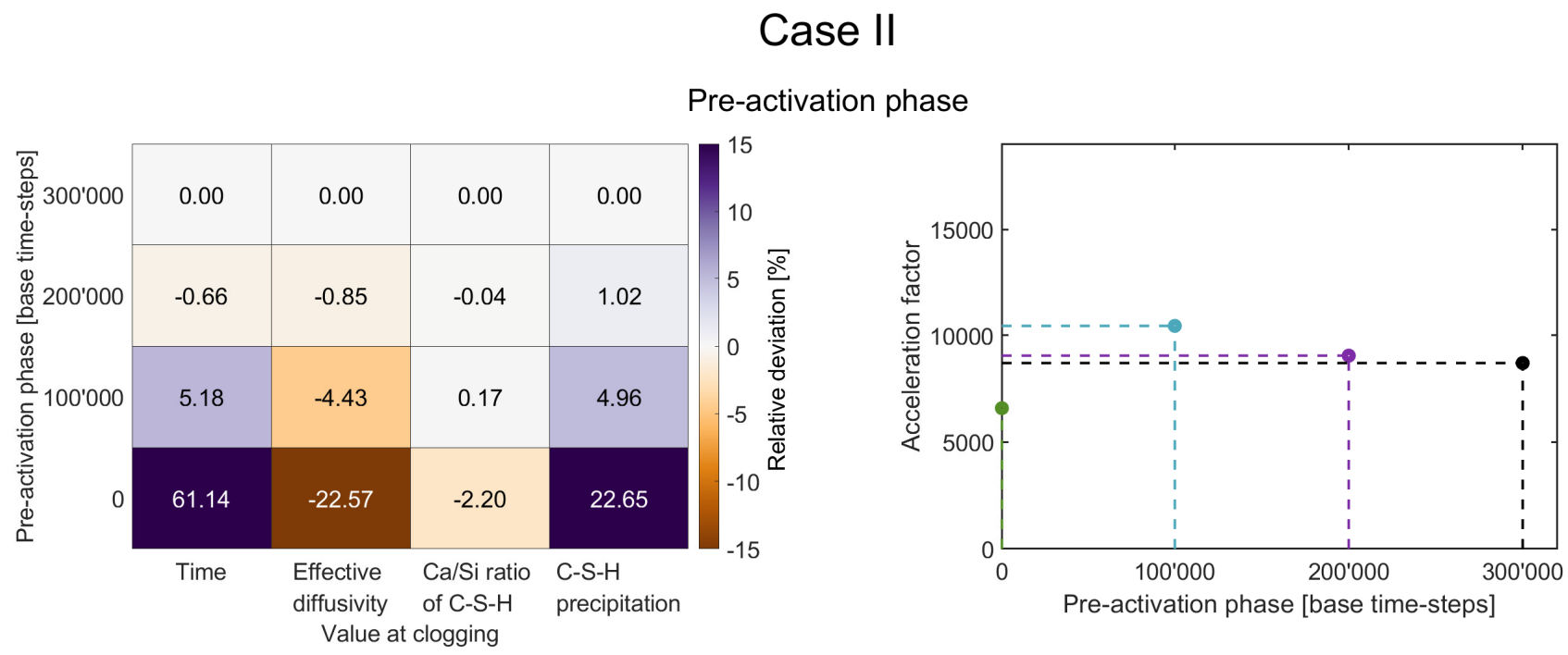

Fig. 6 Heatmap with the relative deviations compared to the simulation without the adaptive time-stepping algorithm for the simulation set of varying the pre-activation phase for relevant parameters at clogging (left) for Case II. Plot for the acceleration factors (right).

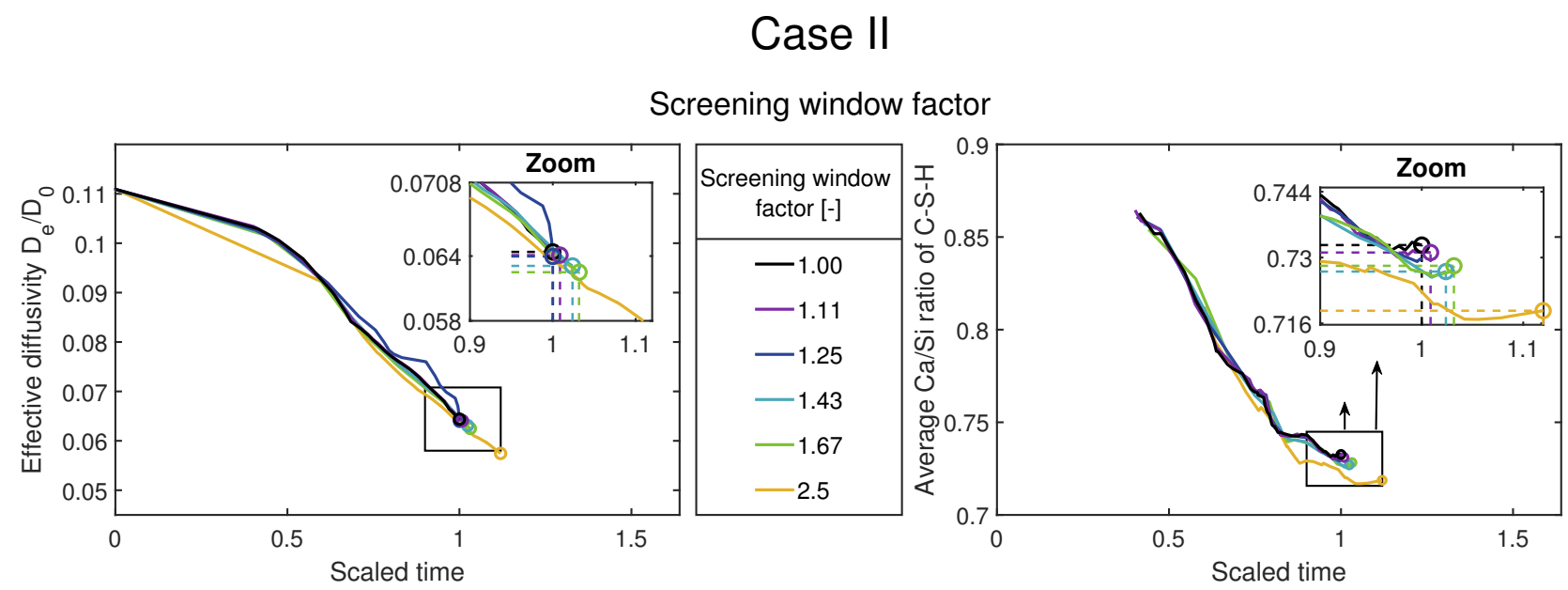

Fig. 7 Time evolution of the effective diffusivity (left) and Ca/Si ratio of C-S-H (right) for all simulations of varying the screening window factor for Case II.

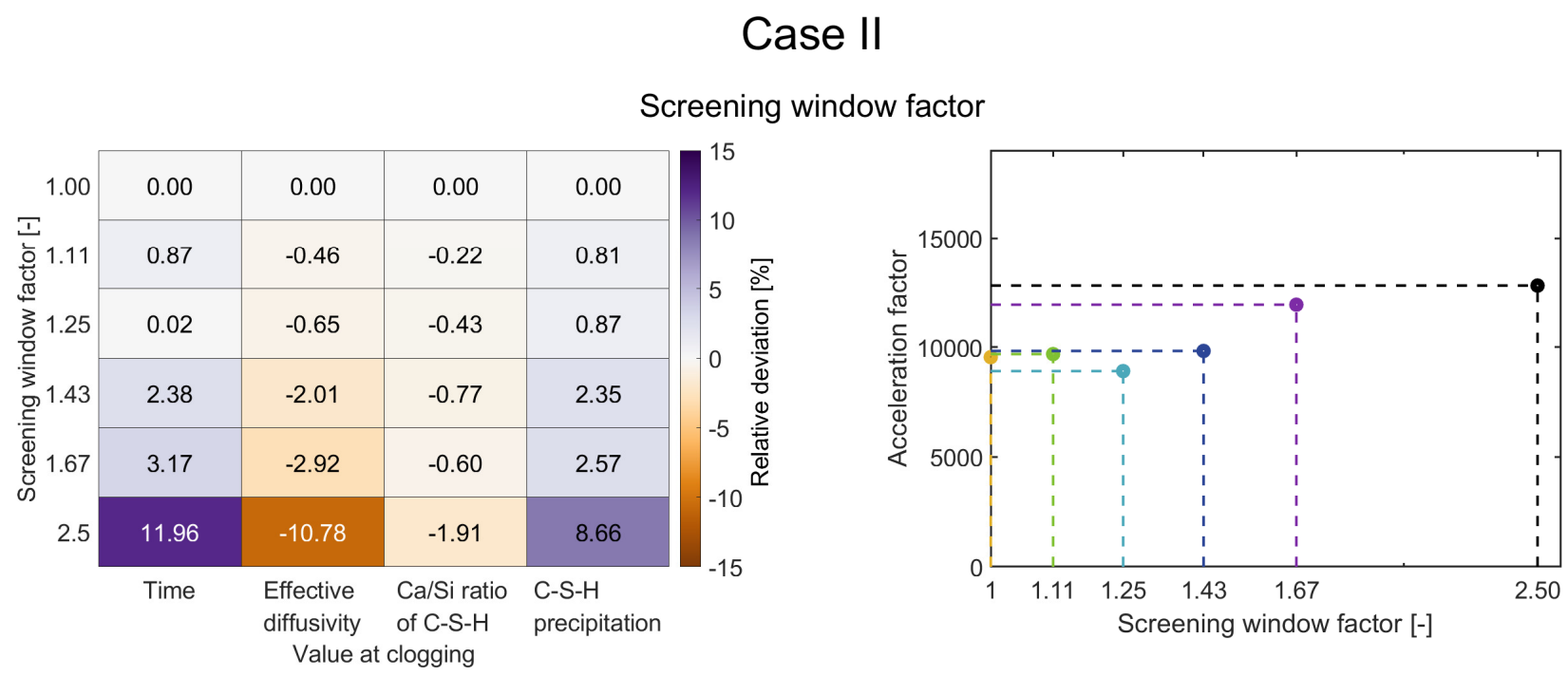

Fig. 8 Heatmap with the relative deviations compared to the simulation without the adaptive time-stepping algorithm for the simulation set of varying the screening window factor for relevant parameters at clogging (left) for Case II. Plot for the acceleration factors (right).

Table 4 Setup of the three cases used to test the adaptive time-stepping algorithm for the Supplementary Case.

	Supplementary Case
Simulation domain size in μm	8 x 4.70
Simulation domain size in voxels	153 x 90
Resolution in μm	0.052
Initial porosity	0.34
Gel pores during RT-simulation	closed
Gel pores during diffusion simulation	open

Table 5 Boundary and initial conditions for the Supplementary Case.

	Left boundary	Right boundary	Initial bulk
$Ca(OH)_{2\,[aq]}$	Fixed at 2.4 mmol L^{-1}	Neumann (zero rate of change)	0.5 mmol L^{-1}
$SiO_{2\,[aq]}$	Fixed at 0.25 mmol L^{-1}	Fixed at 1.7 mmol L^{-1}	1.7 mmol L^{-1}

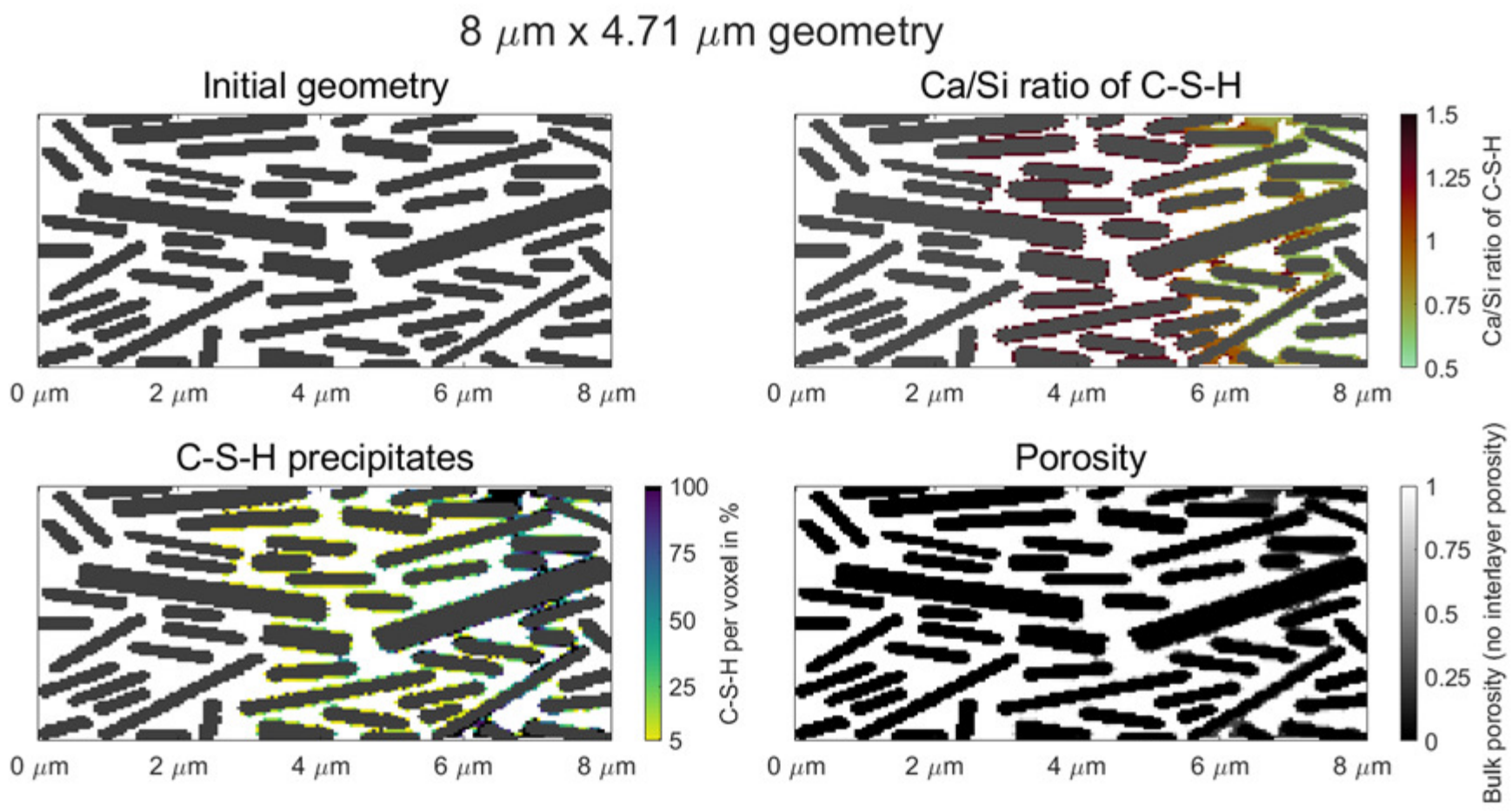

Fig. 9 Initial geometry of porous media for the supplementary case with the 8 µm x 4.71 μm simulation domain (top, left) and the ones close to clogging conditions with information about Ca/Si ratio of C-S-H (top, right), C-S-H precipitates (bottom, left) and porosity (bottom, right). It is the simulation domain without applying the adaptive time-stepping algorithm.

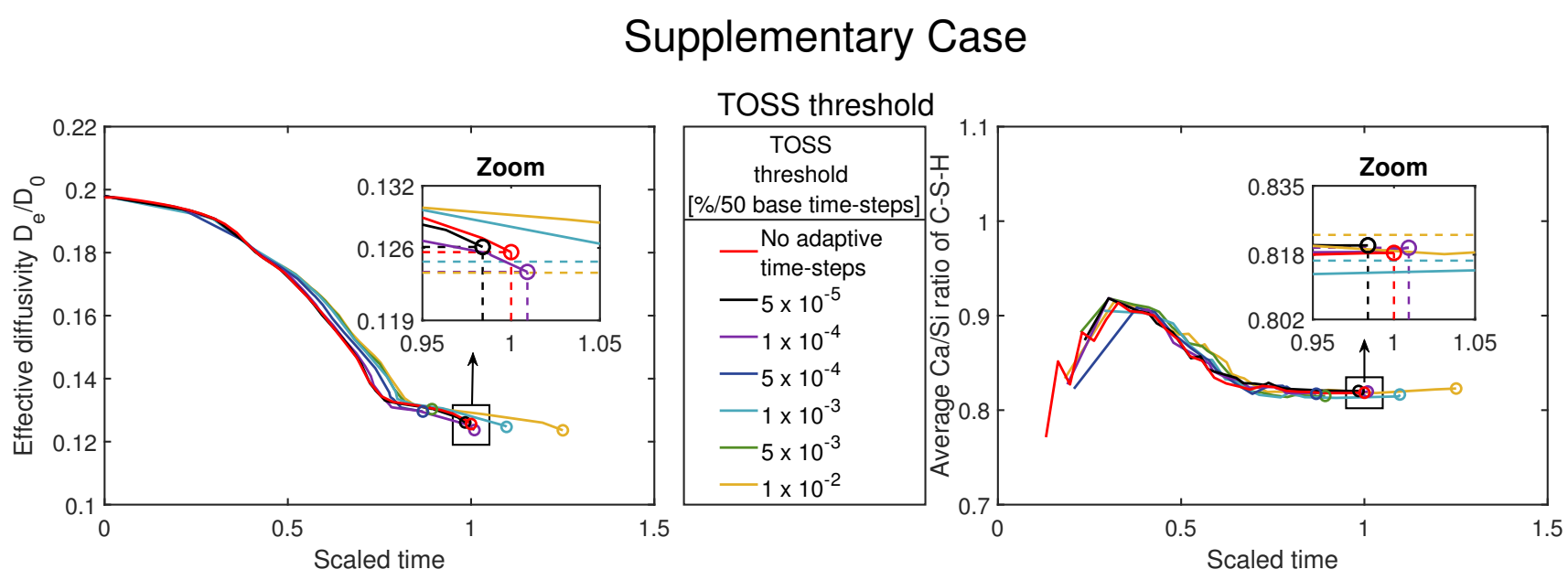

Fig. 10 Time evolution of the effective diffusivity (left) and Ca/Si ratio of C-S-H (right) for all simulations of varying the TOSS threshold forthe Supplementary Case.

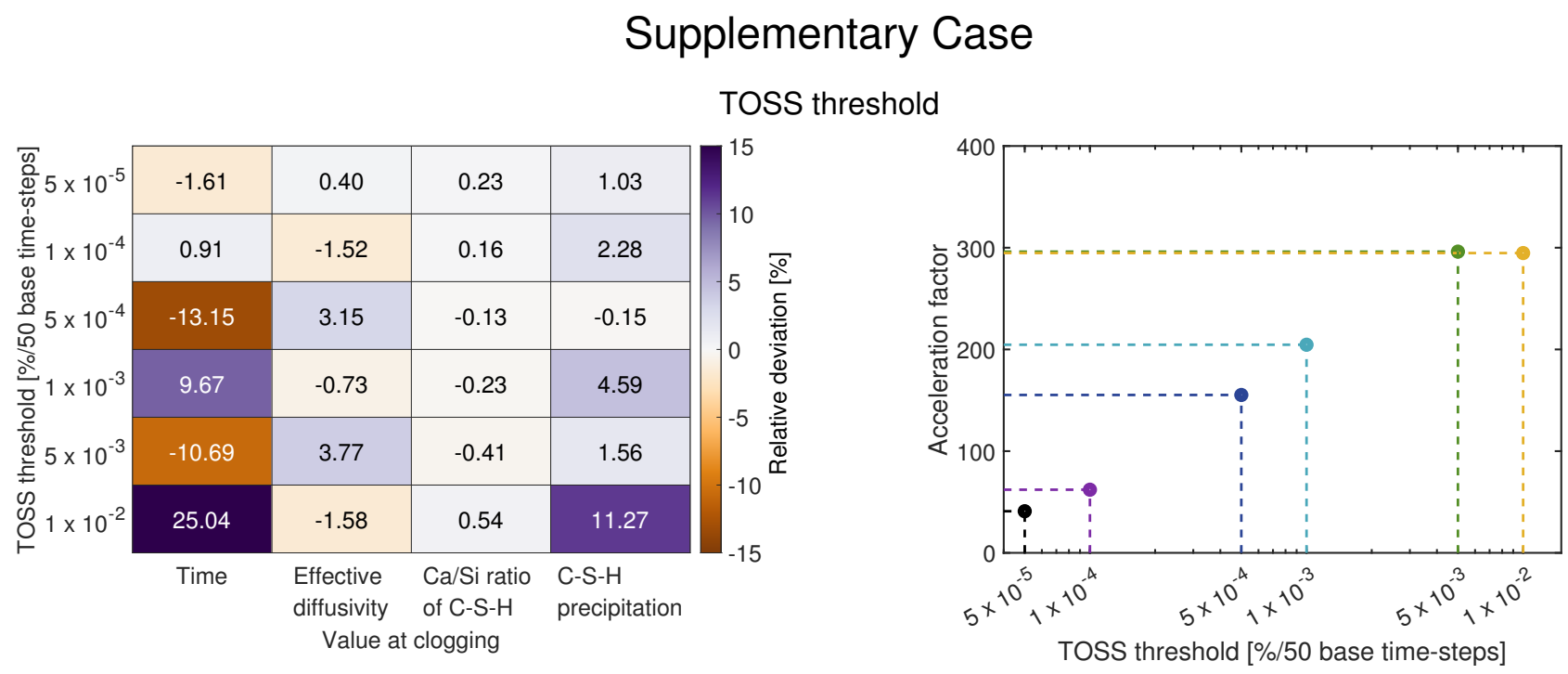

Fig. 11 Heatmap with the relative deviations compared to the simulation without the adaptive time-stepping algorithm for the simulation set of varying the TOSS threshold for relevant parameters at clogging (left) for the Supplementary Case. Plot for the acceleration factors (right).

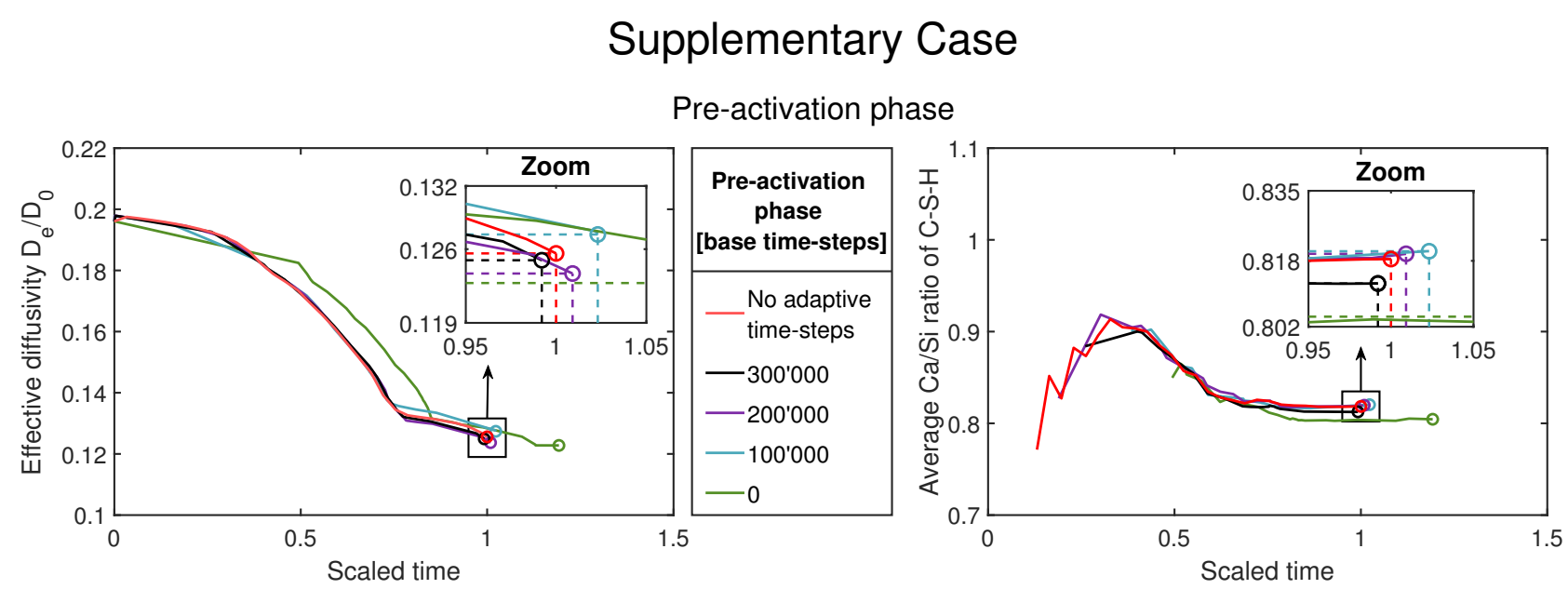

Fig. 12 Time evolution of the effective diffusivity (left) and Ca/Si ratio of C-S-H (right) for all simulations of varying the pre-activation phase for the Supplementary Case.

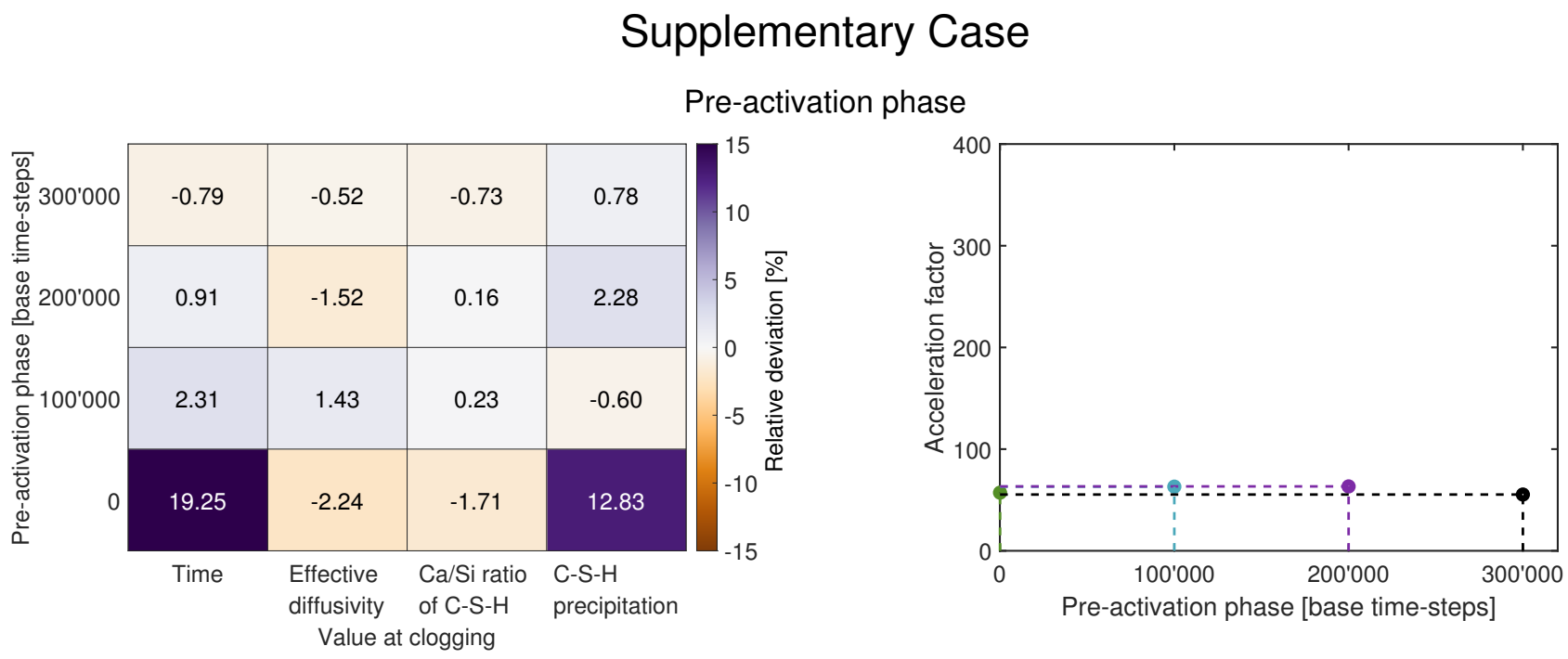

Fig. 13 Heatmap with the relative deviations compared to the simulation without the adaptive time-stepping algorithm for the simulation set of varying the pre-activation phase for relevant parameters at clogging (left) for the Supplementary Case. Plot for the acceleration factors (right).

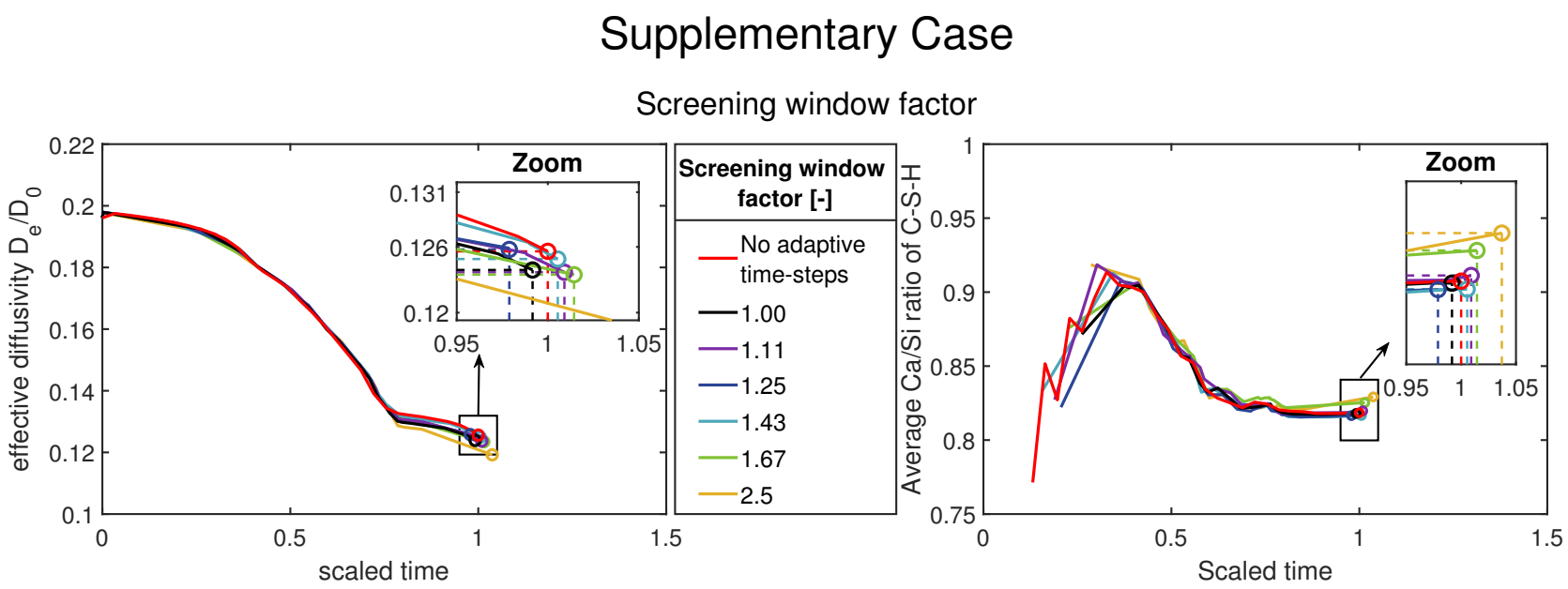

Fig. 14 Time evolution of the effective diffusivity (left) and Ca/Si ratio of C-S-H (right) for all simulations of varying the screening window factor for the Supplementary Case.

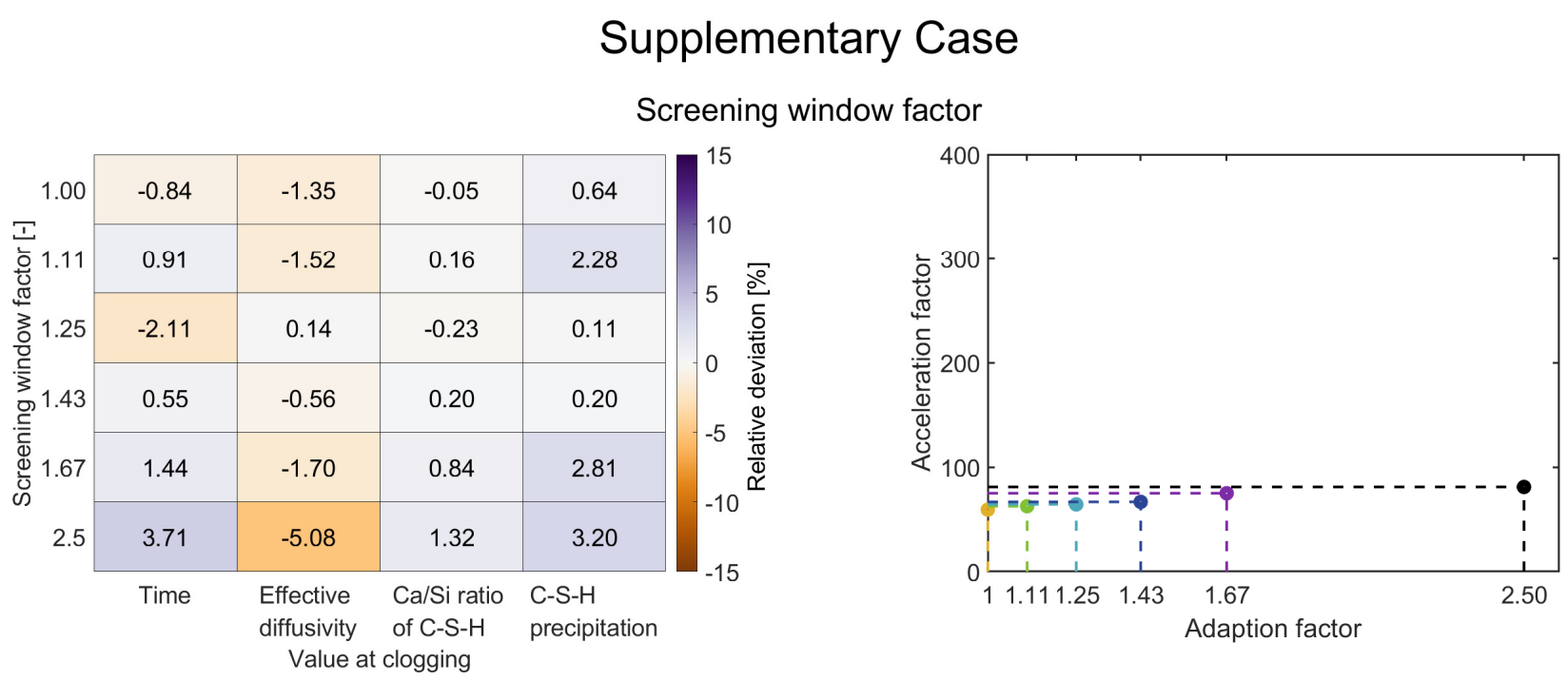

Fig. 15 Heatmap with the relative deviations compared to the simulation without the adaptive time-stepping algorithm for the simulation set of varying the screening window factor for relevant parameters at clogging (left) for the Supplementary Case. Plot for the acceleration factors (right).

5 Calculation of reaction rates per voxel

The average reaction rates are calculated for the voxels in the reactive zone of both simulation domains by dividing the precipitation per time-step and voxel by the surface area of one voxel (one flat surface of a cube side of the voxel for simplification) and the time-step.

The time conversion factor T_{conv} is calculated after(**?**) with the length per voxel L_{Real} which depends on the simulation domain, the voxel length in Lattice-Boltzmann units $L_{\mathrm{LB}} = 1$ lu, the real diffusivity $D_{\mathrm{Real}} = 10^{-11}$ m^2 s^{-1} and the diffusivity in Lattice-Boltzmann units $D_{\mathrm{LB}} = 1/6$. The real time T_{Real} of a time-step is calculated by multiplying T_{conv} with the Lattice-Boltzmann time per time-step T_{LB} which corresponds to 1 lu.

$$T_{\mathrm{conv}} = \frac{L_{\mathrm{Real}}}{L_{\mathrm{LB}}^2} \cdot \frac{D_{\mathrm{LB}}}{D_{\mathrm{Real}}} \tag{3}$$

The reaction rate is calculated for each case with two different surface areas. First, a flat surface of the voxel is considered to account for the lowest possible surface area (resulting in the highest reaction rate).

In Case I, the 2 x 0.86 µm simulation domain with $L_{\mathrm{Real}} = 1.72 \times 10^{-8}$ m results in a base time-step of 4.95×10^{-6} s and a surface area of 3.08×10^{-6} m^2. The simulations are characterized by an average precipitation of 4.00×10^{-10} m^3 l^{-1} per voxel and time-step. This corresponds to 7.7×10^{-21} mol s^{-1} while considering an inverse molar volume of 18'200 m^3 mol^{-1} (value of Tob-H) and a voxel volume of 5.40×10^{-24} l. This results in an average reaction rate of 2.6×10^{-5} mol s^{-1} m^{-2} per voxel in the reactive zone with the equilibrium approach.

In Case II and III, the 10 x 6.25 µm simulation domain with $L_{\mathrm{Real}} = 3.32 \times 10^{-8}$ m results in a base time-step of 2.87×10^{-5} s and a surface area of 1.72×10^{-15} m_2. The simulations are characterized by an average precipitation of 4.00×10^{-12} m^3 l^{-1} per voxel and time-step. This corresponds to 1.8×10^{-22} mol s^{-1} while considering an inverse molar volume of 18'200 m^3 mol^{-1} (value of Tob-H) and a voxel volume of 7.23×10^{-23} l. This results in an average reaction rate of 1.1×10^{-7} mol s^{-1} m^{-2} per voxel in the reactive zone with the equilibrium approach.

In a second approach, the surface area of C-S-H in a voxel completely filled with C-S-H is considered for the highest possible surface area (resulting in the lowest reaction rate). Eq. 4 from (**?**) calculates the specific surface area in m_2 g^{-1}, which is multiplied by the molar mass of C-S-H (in average 130 g mol^{-1}) to get the specific surface area in m^2 mol^{-1}. This value is then multiplied by the molar inverse of 18'200 m^3 mol^{-1} (value of Tob-H) and the volume of a voxel (case dependent) to obtain the absolute surface per voxel. This value is used for calculating the reaction rate like in the first approach.

$$\mathrm{SSA}_{\text{C-S-H}} = 269.3 - 325.1 \log_{10}\left(\left(\frac{\mathrm{Ca}}{\mathrm{Si}}\right)_{\text{C-S-H}}\right) \tag{4}$$

In Case I, an average Ca/Si ratio of C-S-H of 0.9 leads to $\mathrm{SSA}_{\text{C-S-H}} = 284.2$ m^2 g^{-1} according to Eq. 4. Following the previous description, it leads to a reaction rate of 2.2×10^{-6} mol s^{-1} m^{-2} per voxel in the reactive zone.

In Case II and III, an average Ca/Si ratio of C-S-H of 0.8 leads to $\mathrm{SSA}_{\text{C-S-H}} = 300.8$

m^2 g^{-1} according to Eq. 4. Following the previous description, it leads to a reaction rate of 3.6 x 10^{-9} mol s^{-1} m^{-2} per voxel in the reactive zone.

References

Patel, R.A., Perko, J., Jacques, D., De Schutter, G., Van Breugel, K., Ye, G.: A versatile pore-scale multicomponent reactive transport approach based on lattice Boltzmann method: Application to portlandite dissolution. Phys. Chem. Earth **70-71**, 127–137 (2014) https://doi.org/10.1016/j.pce.2014.03.001

Suda, Y., Saeki, T., Saito, T.: Relation between chemical composition and physical properties of c-s-h generated from cementitious materials. Journal of Advanced Concrete Technology **13**, 275–290 (2015) https://doi.org/10.3151/jact.13.275
